# Controlled drug release and electroconductive performance of 3D printed scaffolds for neural tissue regeneration

**DOI:** 10.1007/s10856-026-07043-0

**Published:** 2026-04-25

**Authors:** Busra Oktay, Fatih Ciftci, Israa F. Abdulazez, Ayse Betul Bingol, Alpay Kose, Azime Erarslan, Cem Bulent Ustundag

**Affiliations:** 1 Department of Bioengineering, Faculty of Chemical and Metallurgical Engineering, Yildiz Technical University, Istanbul, Türkiye; 2 Department of Biomedical Engineering, Fatih Sultan Mehmet Vakif University, Istanbul, Türkiye; 3 Biomedical Electronic Design Application and Research Center (BETAM), Fatih Sultan Mehmet Vakıf University, Istanbul, Türkiye; 4 BioriginAI Research Group, Department of Biomedical Engineering, Fatih Sultan Mehmet Vakıf University, Istanbul, Türkiye; 5 Biomedical Engineering Department, Al-Khwarizmi College of Engineering, University of Baghdad, Baghdad, Iraq; 6 Health Biotechnology Joint Research and Application Center, (SABIOTEK), Yildiz Technical University, Istanbul, Türkiye

## Abstract

Nerve cell repair is a complex process influenced by genetic factors, damage severity, and treatment type. Although nerve tissue engineering has advanced, many scaffolds still fail to mimic the natural electrical properties of nerve tissue or deliver drugs effectively. To address these issues, this study presents a multifunctional scaffold designed to support nerve regeneration while reducing inflammation and pain. The scaffold was fabricated using 3D microextrusion printing, allowing precise control over geometry and composition. Polyvinyl alcohol (PVA) and collagen (Col) provided biocompatibility and biodegradability, while reduced graphene oxide (rGO) enhanced electrical conductivity. Amoxicillin (Amox) and ibuprofen (Ibu) were incorporated for antibacterial and anti-inflammatory effects. The scaffold exhibited a conductivity of (5.83 ± 0.65) × 10⁻³ S/m, and sustained drug release, with amoxicillin reaching ~0.6 mg/L and ibuprofen ~0.12 mg/L after 480 min. It showed strong antibacterial activity, with inhibition zones of 28.3 ± 3.32 mm (*E. coli*) and 18.34 ± 2.83 mm (*S. aureus*). Mechanically, it withstood ~5.5 MPa of stress and over 150% tensile strain. Cell viability exceeded 120%, indicating excellent biocompatibility. These results suggest the scaffold effectively integrates conductivity, structural strength, and therapeutic delivery to promote nerve regeneration.

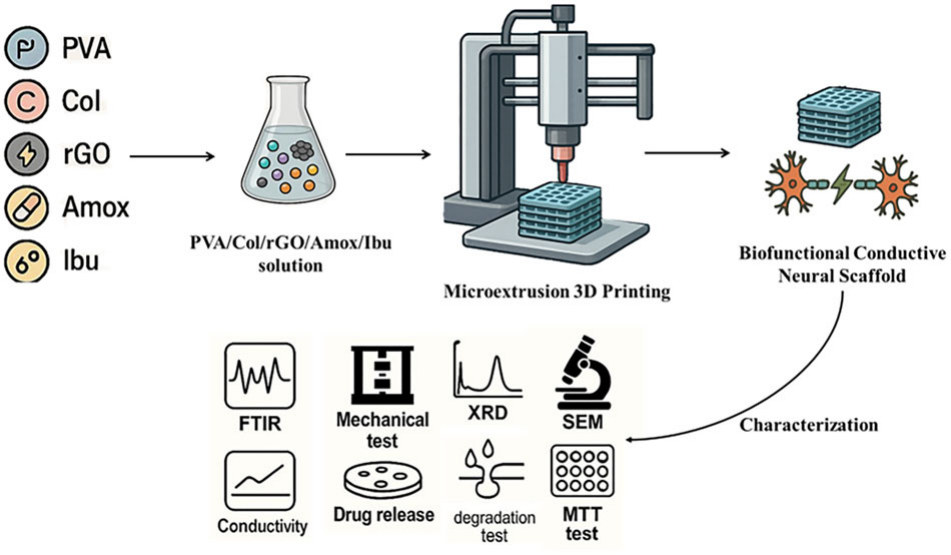

## Introduction

Neural injuries are common conditions resulting from damage or severance of nerve fibers, leading to impaired nerve transmission [1]. Nerves possess a limited regenerative capacity; while minor injuries can heal spontaneously, severe injuries require surgical intervention. Despite advancements in microsurgical techniques, the success rates of nerve repair remain relatively low, with satisfactory outcomes observed in fewer than 40% of cases [2]. The limited regenerative capacity of nerves and the low success rates of surgical approaches highlight a critical unmet clinical need for more effective, targeted strategies that can restore nerve function and accelerate healing [2]. Consequently, bioengineering strategies focus on developing alternatives to nerve grafting and microsurgery to enhance nerve regeneration. Therefore, the primary aim of this study is to design and characterize a multifunctional scaffold that can simultaneously support nerve regeneration, restore electrical signaling, and reduce inflammation and pain in nerve injuries [3]. Neural tissue engineering aims to restore nerve function by facilitating tissue repair and regeneration, offering innovative and effective alternatives to conventional treatment approaches [4–6]. A fundamental strategy in neural tissue engineering involves the fabrication of polymeric scaffolds that provide a supportive three-dimensional (3D) environment for nerve cells to adhere, proliferate, and differentiate [7, 8]. These scaffolds serve as structural frameworks that promote cellular interactions, facilitating functional recovery of the damaged tissue [9, 10]. By integrating conductive and therapeutic components into the scaffold structure, this approach directly addresses the limitations of conventional scaffolds that lack electrical functionality and local drug delivery capacity [11]. In this context, biofunctional and conductive neural scaffolds are designed to mimic the structural and functional properties of native neural tissue, supporting cell adhesion, proliferation, and communication. These scaffolds not only provide mechanical support but also integrate conductive materials to enhance electrical signaling, which is crucial for nerve tissue [12–14]. By incorporating conductive biomaterials, biofunctional scaffolds can potentially improve the efficiency of neural regeneration, making them promising candidates for nerve repair applications [15, 16]. In this study, customized scaffolds were designed using polyvinyl alcohol (PVA), a biocompatible polymer, via 3D microextrusion printing [17]. PVA is widely utilized in scaffold fabrication due to its biocompatibility, biodegradability, and ability to reduce inflammatory responses in nerve injuries [18–21]. Collagen (Col), a natural protein abundantly found in various tissues, plays a crucial role in mimicking the microenvironment of neural tissue, providing structural support and contributing to wound healing [22, 23]. The surface properties of PVA/Col scaffolds facilitate cell adhesion, proliferation, and neural tissue regeneration, making them promising biomaterials for nerve repair. The selection of bioactive materials that support nerve regeneration is a fundamental aspect of neural tissue engineering [24, 25]. In this regard, conductive scaffolds are particularly important for tissue engineering applications that require electrical activity, such as nerve and muscle tissue regeneration. These scaffolds facilitate the transmission of electrical signals and help maintain electrical activity within the tissue, thereby promoting functional recovery [26–28]. By mimicking the natural electrical properties of neural tissue, conductive scaffolds hold great potential for applications in nerve repair and muscle tissue regeneration. Scaffolds for tissue engineering must be fabricated from biocompatible and body-compatible materials to ensure successful integration and functionality [22, 29]. The incorporation of conductive nanomaterials, such as reduced graphene oxide (rGO), enhances electrical communication within the scaffold, which is crucial for neural tissue engineering [30–33]. Additionally, the controlled release of therapeutic agents through scaffolds has the potential to accelerate the healing process. The controlled delivery of antibiotics and anti-inflammatory drugs aims to reduce the risk of infection and regulate inflammation, while analgesics contribute to patient comfort and facilitate recovery [34, 35]. Peripheral nerve injuries are frequently associated with post-traumatic inflammation and high susceptibility to bacterial contamination, particularly in open or surgical lesions [36]. It has been reported that excessive inflammation delays Schwann cell migration and axonal extension and negatively influences functional recovery, highlighting the importance of early anti-inflammatory intervention in neural repair [37]. Therefore, amoxicillin was specifically selected as a broad-spectrum β-lactam antibiotic to minimize early-stage bacterial colonization and reduce infection-driven inflammation in the regenerating neural microenvironment against both Gram-positive and Gram-negative bacteria, which are commonly encountered in wound environments [38]. Ibuprofen was incorporated as a non-steroidal anti-inflammatory drug (NSAID) to modulate inflammatory responses and reduce prostaglandin-mediated pain, which can otherwise impair axonal regeneration [39, 40]. Importantly, previous studies have demonstrated that local ibuprofen delivery in peripheral nerve injury models can enhance axonal growth and improve functional outcomes by modulating inflammatory cascades [40, 41]. Thus, the combined incorporation of amoxicillin and ibuprofen was strategically designed to address two critical barriers to nerve regeneration: early infection risk and acute inflammatory response, while simultaneously improving patient comfort through analgesic effects [37]. This dual approach aligns with contemporary regenerative medicine strategies that emphasize localized, multifunctional therapeutic modulation within implantable scaffolds.

Three-dimensional printing technologies offer significant advantages in tissue engineering by enabling the fabrication of highly customized scaffolds with adjustable porosity, mechanical strength, and complex architectures that promote cell growth and tissue regeneration [42–44].

Although several studies have reported conductive neural scaffolds or drug-loaded polymeric systems separately, the present study introduces a synergistic multifunctional platform that integrates (i) electroconductive rGO reinforcement, (ii) a hybrid biopolymer–synthetic polymer matrix (PVA/Col), and (iii) a dual-drug delivery system within a precisely controlled microextrusion-printed architecture. Graphene-based composites such as rGO-reinforced polymers have been widely recognized for their ability to improve electrical conductivity, mechanical strength, and cellular interactions in neural tissue engineering, enabling enhanced neurite extension and functional recovery compared to non-conductive materials [45, 46]. The combined incorporation of rGO into the polymer matrix not only enhances electroactivity but also improves scaffold stability and promotes a bioelectric microenvironment that supports neuronal growth and guidance, as demonstrated in recent work on conductive biomaterials for nerve repair [47, 48]. In parallel, the dual loading of amoxicillin and ibuprofen introduces a biologically responsive dimension by simultaneously addressing early-stage bacterial risk and acute inflammatory cascades that may otherwise impair axonal regeneration, aligning with current strategies for controlled therapeutic delivery in regenerative scaffolds [37, 40, 41, 49]. In the literature, the simultaneous combination of rGO-mediated electroactivity and dual Amox/Ibu release within a 3D microextrusion-printed PVA/Col scaffold has not been previously reported for neural tissue engineering applications. This integrated design offers concurrent electrical stimulation capability, mechanical adaptability, and localized therapeutic modulation within a single biomimetic construct, representing a novel and comprehensive approach to neural scaffold development. In this study, the 3D biofunctional and conductive neural scaffolds were developed by incorporating rGO into biocompatible PVA/Col scaffolds, along with anti-inflammatory and analgesic drugs to enhance their biofunctionality. The developed 3D scaffolds were morphologically, chemically, mechanically, and biologically characterized to assess their potential for neural tissue engineering applications. These findings suggest that the developed 3D scaffold system could serve as a viable alternative to current nerve repair strategies, combining structural support, biofunctionality, and electrical conductivity within a single platform.

## Materials and methods

### Materials

Graphene, sulfuric acid (95–97%, Mw: 98.08, Merck, Germany), phosphoric acid (85%, Mw: 98, Merck, Germany), potassium permanganate (Mw: 158.03, ISOLAB, Germany), hydrogen peroxide (30%, Mw: 34.01, Cinnagen, Iran), hydrochloric acid (37%, Mw: 36.46, Merck, Germany), and ethanol (99.5%, Mw: 46.07, Merck, Germany) were used for reduced graphene oxide synthesis. Polyvinyl alcohol (pellet forms, 98–99% hydrolyzed, Mw: 31,000–50,000, Sigma-Aldrich, USA), collagen (powder, from Bovine hide, 97% Type I with the remainder of Type III collagen, Merck, Germany), Amoxicillin (95–102% anhydrous, Mw:365.40, Sigma-Aldrich, USA), and Ibuprofen ((S)-2-(4-Isobutylphenyl)propanoic acid((S)-(+)-Ibuprofen), ≥99.8%, Mw: 206.28 g/mol, BLD pharm, India).

### Preparation of PVA/Col/rGO/Amox/Ibu Solution for 3D Printing

#### Synthesis of reduced graphene oxide

In this experiment, graphene oxide (GO) was synthesized using Hummer’s method. Initially, 360 mL of sulfuric acid (H₂SO₄) and 40 mL of phosphoric acid (H₃PO₄) were added to a beaker. The beaker was then placed on a silicone oil bath to maintain a consistent temperature, with the oil heated to 55–60 °C. This temperature range kept the reaction temperature stable between 40 and 45 °C. The beaker, along with the oil bath, was placed in a mixer and stirred at 200 rpm. Subsequently, 3 g of graphite and 18 g of potassium permanganate (KMnO₄) were gradually added to the solution, which was stirred at 40–45 °C for 16 h. After 16 h of stirring, the reaction was stopped by transferring the solution to a beaker containing 400 g of ice. During the mixing process, 7 mL of hydrogen peroxide (H₂O₂) was added drop by drop, causing the solution to turn yellow. For the washing step, the solution was first precipitated, then centrifuged (Nuve, NF 800, Turkey) at 6000 rpm for 45 min: first with water, followed by three washes with hydrochloric acid (HCl), and then three washes with ethanol. Finally, the material was dried in an oven at 60 °C (Elektro-mag, M420P, Turkey) [50]. To reduce graphene oxide, the oxygen-containing functional groups were removed to restore its electrical conductivity. The reduction was achieved through thermal treatment. 1 g of GO powder was wrapped in aluminum foil, ensuring it was tightly sealed on all sides. The wrapped powder was then placed in a preheated muffle furnace (Liya Test, LT-G0275, Türkiye) set to 350 °C, where it was treated thermally for 1 min. It was essential to gradually remove the crucible from the furnace to prevent any material loss due to abrupt pressure changes, which could cause the material to disperse into the air. The final product was a black powder [30, 51].

#### Preparation of PVA/Col/rGO/Amox/Ibu solution

**Fig. 1:**
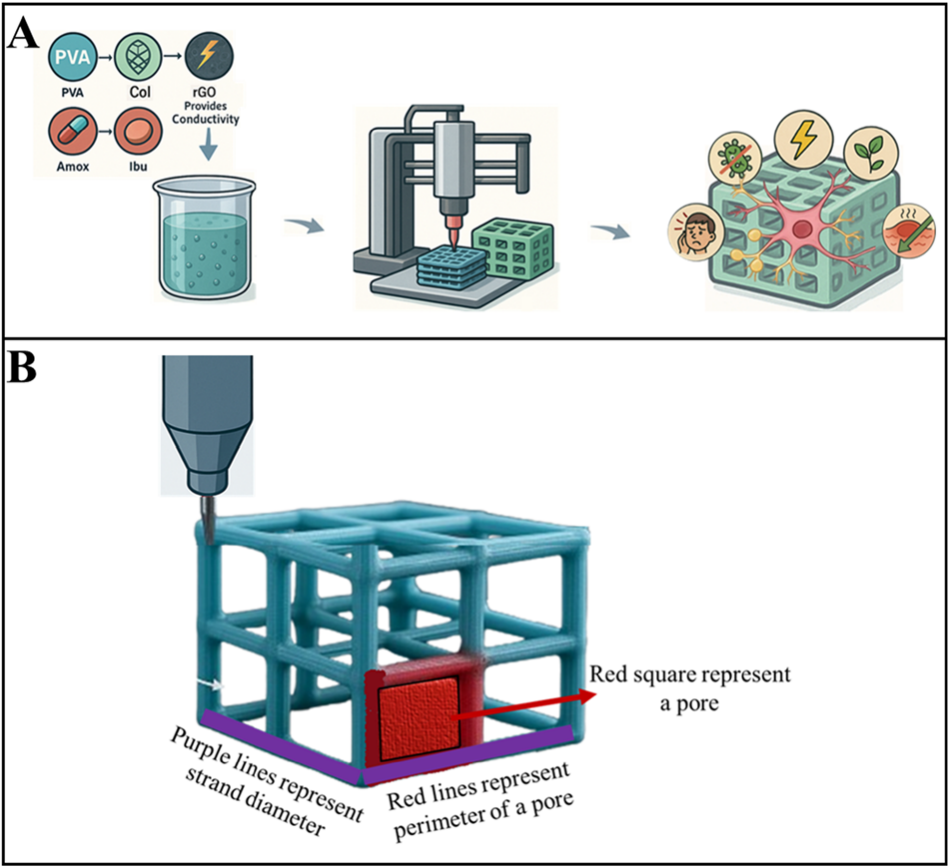
**A** Experimental part of the 3D biofunctional and conductive neural scaffolds. **B** Printability assessment of the 3D biofunctional and conductive neural scaffold

In this study, PVA, Col, rGO, and drugs were combined to fabricate a conductive scaffold via microextrusion 3D printing (Fig. 1A). PVA and collagen are water-soluble polymers [52, 53]. A 20% (w/w) PVA/Col (9:1) solution was mixed in 10 ml of distilled water at 70 °C until homogeneous. To enhance the conductivity properties of the scaffold, rGO was added, while Amoxicillin (Amox) was added to improve anti-inflammatory properties, and Ibuprofen (Ibu) was included to provide analgesic effects. The amounts of rGO, Amox, and Ibu added to the PVA/Col solution were each 1% of the total polymer weight, as shown in Table 1.

**Table 1:** Amounts of biomaterials used in neural scaffolds

		PVA	Col	rGO	Amox	Ibu
PVA/Col	9:1	1.8 g	0.2 g	–	–	–
PVA/Col/rGO	90:10:1	1.8 g	0.2 g	0.02 g	–	–
PVA/Col/rGO/Amox/Ibu	90:10:1:1:1	1.8 g	0.2 g	0.02 g	0.02 g	0.02 g

### Neural tissue fabrication by 3D printing

3D scaffolds were produced using a microextrusion printing device (AXO-MEW, Turkey) with an X-Y control head and a heated substrate that moves in the Z-direction to build the structure layer by layer. The 3D printing was carried out under room temperature conditions. The biofunctional and conductive scaffold was designed using SolidWorks as the Computer-Aided Design (CAD) program. The CAD models were subsequently converted into STL file format, a technology designed for the production of digital 3D structures. To fabricate 3D scaffolds, the PVA/Col/rGO/Amox/Ibu solution was extruded through a syringe needle with the assistance of pneumatic pressure, carefully depositing the material layer by layer onto the surface. The rheological behavior of the printing ink was visually assessed to ensure continuous filament formation without bead formation or collapse. Different 3D printing parameters used for optimization were given in Table 2. During the optimization of the production process, 25-Gauge needle tips were used. The production was completed with a flow rate of 5 mm/s, a feed rate of 30%, a pressure of 28–30 psi, an extrusion multiplier of 45, an infill density of 46%, and a layer thickness of 0.1 mm, resulting in scaffold dimensions of 30 × 30 mm. The optimized parameters were chosen to balance mechanical integrity and pore interconnectivity, which are critical for nutrient diffusion in neural tissue applications.

**Table 2:** Different 3D printing parameters used for optimization

PVA (w/v)	Needle tip (Gauge)	Flow rate (mm/s): Feed rate (%)	Pressure (psi)	Infill density (%)	Number of layers
16%	27	1:25	12–16	22	5
17%	27	1:30	15	28	5
17%	27	1:30	15	32	4
18%	25	5:20	16–18	22	4
18%	25	5:20	16–18	32	7
18%	25	5:20	16–18	46	4
20%	25	5:50	28–30	22	5
20%	25	5:30	28–30	46	5
20%	25	5:30	28–30	46	7

### Post-indentation characterization of nanomaterials through Raman spectroscopy

The Raman spectrometer enables a detailed analysis of the material’s properties post-indentation, offering insights into alterations in molecular structure, mechanical behavior, and surface characteristics. Post-indentation characterization of the samples (GO and rGO) was conducted using a Renishaw 1000 Raman spectrometer, equipped with a load-coupled device detector and a microscope that focused a laser beam on a 1 mm spot size.

### Chemical characterization of the 3D biofunctional and conductive neural scaffolds through FTIR spectroscopy

Fourier Transform Infrared (FTIR) Spectroscopy is a technique used to analyze the chemical composition and functional groups of a material by measuring how it absorbs infrared light. This method provides valuable information about the material’s molecular structure and chemical bonds. FTIR spectroscopy was employed to chemically characterize the samples (PVA, Col, GO, rGO, Amox, Ibu) and the developed 3D scaffolds (PVA/Col, PVA/Col/rGO, PVA/Col/rGO/Amox/Ibu) using an IR Affinity-1 infrared spectrophotometer (Jasco, FT/IR-4700 type A) across a spectrum range of 400–4000 cm^−1^.

### Crystallinity analysis of the 3D biofunctional and conductive neural scaffolds via XRD

X-ray Diffraction (XRD) is a method used to analyze the crystalline structure, phase composition, and grain size of materials by measuring the diffraction patterns produced when X-rays interact with the atomic lattice. This technique provides crucial information about the structural properties of the 3D scaffolds (PVA, PVA/Col, PVA/Col/rGO, PVA/Col/rGO/Amox/Ibu). The crystal structures of the developed 3D scaffolds were analyzed using XRD (Shimadzu-6100, Japan) with a Cu-Kα radiation source (λ = 1.54060 Å). The diffraction patterns were recorded over a 10–80° angle range using a 40-mA current and a 45 kV voltage generator.

### Measurement of the Conductivity of the 3D Biofunctional and Conductive Neural Scaffolds using the Four-Point Probe Method

The four-point probe method is used to measure sheet resistance and was performed using a four-point probe with equally spaced, co-linear electrical probes. It operates by applying a Direct-Current (DC) (I) between the two outer probes and measuring the voltage drop between the two inner probes. Samples measured by the four-point probe method are required to have no porous structure [54]. Therefore, to determine the conductivity of the developed 3D scaffolds (PVA, PVA/Col, PVA/Col/rGO, PVA/Col/rGO/Amox/Ibu), the PVA-based solutions were cast onto a polytetrafluoroethylene (PTFE) disk separately. The disks were placed in a vacuum dryer and left to dry at room temperature for 12 h to eliminate pores. The obtained films were cut into 5 mm strips for conductivity measurement. The cut strips were placed onto the electrodes. The conductivity measurement of the films was conducted using a potentiostat and the four-point probe (Signatone, Pro4, USA). The measurements were repeated three times. For conductivity, the resistivity (ohm*cm) is converted to siemens/meter (S/m) [55, 56].

### High-resolution imaging and structural analysis of nanoparticles with TEM

Transmission Electron Microscopy (TEM) is a high-resolution imaging technique used to analyze nanomaterials, biomaterials, and polymers. The structural analysis of GO and rGO nanoparticles was performed using a TEM (JEOL JEM-2100, Japan) operating at an accelerating voltage of 200 kV. For TEM imaging, dilute aqueous dispersions of GO and rGO were prepared by ultrasonic treatment, and each sample was dropped onto carbon-coated copper grids. The grids were dried at room temperature before imaging.

### Printability assessment of the 3D biofunctional and conductive neural scaffolds

Printability formulas are used to evaluate the ability of scaffolds (PVA, PVA/Col, PVA/Col/rGO, PVA/Col/rGO/Amox/Ibu) to be stably printed using a 3D printer. Mathematical formulas are applied to define the printability (Pr) of the ink (Fig. 1B) [57].

1 $${\mathrm{Strand}}\,{\mathrm{Printability}}=\frac{D\,exp}{Ds}* 100$$

Ds is the standard strand diameter, and Dexp is the experimental strand diameter in Eq. (1). This formula calculates the printability of the strand by comparing the standard and experimental diameters. In another assessment, pore printability is defined, where *p* represents the pore perimeter, and β is the pore area in a scaffold (Eq. 2) [57].

2 $${\rm{Pore\; printability}}=\frac{{{\rm{p}}}^{2}}{16{\rm{\beta }}}$$

### Examination of dimensions and layer alignment of the 3D biofunctional and conductive neural scaffolds using optical microscopy

The optical images of the developed 3D scaffolds (PVA, PVA/Col, PVA/Col/rGO, PVA/Col/rGO/Amox/Ibu) were captured using a Digital Microscope (BOROX, DIJISKOP7, China) at a 500× magnification. Each sample was measured with a ruler and had an area of 30*30 mm². Based on the measurements, the printability values of the scaffolds were calculated using Eqs. (1) and (2).

### Investigation of the surface morphologies of the 3D biofunctional and conductive neural scaffolds via SEM

Scanning Electron Microscopy (SEM) is a technique employed for investigating the surface morphology, topography, and microstructural characteristics of materials by scanning the surface, offering high-resolution imaging for detailed analysis. For examining the surface morphology of the developed 3D scaffolds (PVA, PVA/Col, PVA/Col/rGO, PVA/Col/rGO/Amox/Ibu), SEM (EVA MA 10, ZEISS, USA) was used. Prior to imaging, the surface of the samples was coated with gold for 120 s using a sputter coating machine (Quorum, SC7620, USA), and then the analysis was performed. The samples were mounted onto the surface using double-sided carbon tape for stabilization during SEM analysis.

### Thermal analysis of the 3D biofunctional and conductive neural scaffolds (DSC-TGA)

Differential Scanning Calorimetry (DSC) measures a material’s heat flow response to temperature changes, analyzing the thermal properties of polymers. The thermal properties of the developed 3D scaffolds (PVA, PVA/Col, PVA/Col/rGO, PVA/Col/rGO/Amox/Ibu) were analyzed with DSC and TGA. DSC was carried out at a heating rate of 10 °C/min, in the temperature range of 20–260 °C, under continuous nitrogen gas flow using a Perkin-Elmer DSC 4000 device. Thermogravimetric Analysis (TGA) was performed at a heating rate of 10 °C/min, from room temperature to 700 °C, under a nitrogen atmosphere using a TA Instruments TGA Q50 apparatus. Derivative Thermogravimetry (DTG) curves were also derived from TGA analysis.

### Mechanical strength testing of the 3D biofunctional and conductive neural scaffolds

Mechanical analysis is crucial as it allows for the precise evaluation of a material’s strength, elasticity, and durability. The tensile strength of the developed 3D scaffolds (PVA, PVA/Col, PVA/Col/rGO, PVA/Col/rGO/Amox/Ibu) was determined using a tensile testing machine (SHIMADZU, EZ-LX, Japan). Before the tensile test, each sample was placed onto rectangular-shaped surfaces 50 mm in length and 10 mm in width. The thickness of the developed 3D scaffolds was measured using a high-accuracy digital micrometer (Mitutoyo MTI Corp., USA). The upper and lower parts of each sample were placed horizontally in the appropriate section of the device. During the tensile test, the speed was set to 5 mm/min, and a force of 0.1 N was applied. The measurements were carried out at room temperature (23 °C). Standard percentage error bars were used to obtain the standard deviation.

### Evaluation of the degradation behavior of the 3D biofunctional and conductive neural scaffolds

The degradation test is used to examine the degradation behavior and when biomaterials begin to biodegrade in a living environment. To evaluate the degradation (weight loss) of the developed 3D scaffolds (PVA, PVA/Col, PVA/Col/rGO, PVA/Col/rGO/Amox/Ibu), they were placed in 2 mL of Phosphate-Buffered Saline (PBS) solution (tablet, pH 7.2–7.6, Sigma-Aldrich, St. Louis, USA). For the degradation tests, the solutions containing the scaffolds were incubated in a thermal orbital shaker (BIOSAN TS-100) at 37 °C and 60 rpm for specific time intervals. The scaffolds were removed from the PBS solution and dried at 37 °C for 12 h. Degradation was determined by measuring weight loss (%) using Eq. (3) [58]:

3 $${\rm{Weight\; loss}}( \% )=\left(\frac{W1-W2}{W1}\right)* 100$$

where W_1_ and W_2_ are the weights of the scaffolds before and after degradation, respectively.

### Evaluation of cumulative drug release and release kinetics from 3D biofunctional and conductive neural scaffolds

To evaluate the cumulative drug release from the developed 3D scaffolds, they were placed in 2 mL of PBS solution at pH 7.4. During the drug release experiments, the PBS solutions containing the scaffolds were incubated in a thermal orbital shaker (BIOSAN TS-100) at 37 °C and 60 rpm for specific time intervals. At specified time intervals, 2 mL of PBS solution was withdrawn, and the same volume of fresh PBS solution was added. The collected samples were analyzed in a UV spectrophotometer (Shimadzu UV-3600, Japan) for the cumulative drug release analysis, and the absorbance values were measured at the appropriate wavelength (nm) for each drug [59]. Calibration curves and cumulative drug release graphs for each drug were plotted based on the obtained results. In the release kinetics modeling, the controlled release behavior of the developed 3D scaffold (PVA/Col/rGO/Amox/Ibu) containing amoxicillin and ibuprofen was investigated. This formulation aims to provide a dual-effect therapy by combining the effects of Amox and Ibu to support biological processes in neural tissue. The cumulative release amounts obtained from experimental data were analyzed according to six different kinetic models: Zero-order, First-order, Higuchi, Hixson–Crowell, Korsmeyer–Peppas, and Weibull models. For each model, the regression coefficient (*R*²), slope, and intercept values were calculated, and their effects on the release mechanisms were discussed in detail.

### In vitro antibacterial activity analysis of the 3D biofunctional and conductive neural scaffolds

The antibacterial properties of the developed 3D scaffolds (PVA, PVA/Col, PVA/Col/rGO, PVA/Col/rGO/Amox/Ibu) were investigated against *Escherichia coli* (*E.coli)* (gram-negative) and *Staphylococcus aureus* (*S.aureus*) (gram-positive) and bacteria using the disk diffusion technique. The pathogens, whose antibacterial activity was assessed, were obtained from the Food Engineering Department Laboratory at Yıldız Technical University. *E.coli* and *S.aureus* bacteria were cultured at 37 °C for 24 h. 15 mL of Muller-Hinton agar (Merck) was given to each infected Petri dish. Then, 0.01 mL of the culture medium was injected into sterilized Petri dishes. All the samples were formed into disks of almost the same size and gently pressed to fix them onto the solid agar medium. The treated Petri dishes were incubated at 37 ± 1 °C for 24 h. The inhibitory zones developed on the medium were finally measured. Antibacterial activity experiments were performed in duplicate for each test strain, and average measurements were calculated.

### In vitro cytocompatibility evaluation of the 3D Biofunctional and conductive neural scaffolds

The biological characterization of the developed 3D scaffolds was conducted using the L929 mouse fibroblast cell line. Before the experiment, all specimens (PVA, PVA/Col, PVA/Col/rGO, PVA/Col/rGO/Amox/Ibu) underwent UV sterilization for one hour and were subsequently placed into 96-well plates. L929 cells were seeded into each well at a 1 × 10^4^ cells/mL density on the prepared scaffolds. The scaffolds were then incubated with 5% CO_2_ at 37 °C for 14 days. The growth medium employed consisted of DMEM- low glucose (Dulbecco’s Modified Eagle’s Medium-low glucose), FBS (fetal bovine serum), penicillin/streptomycin, L-glutamine, and phosphate-buffered saline (PBS) tablets, which were bought from Amresco (Solon, USA). (99% purity, vol./vol.), 3-(4,5-dimethyl-2-thiazol)-2,5-diphenyl-2H-tetrazolium bromide (MTT) powder, Trypsin/EDTA solution at 0.25% (w/v), and dimethylsulfoxide (DMSO) were obtained from Sigma-Aldrich (St. Louis, USA). In vitro cell viability assessment for the L929 mouse fibroblast cells seeded on the scaffolds was performed using the MTT assay on the cell culture’s 1st, 3rd, 5th, and 7th days. The growth medium was removed after incubation at 37 °C with 5% CO_2_ for each predetermined day. Subsequently, 90 µL of fresh medium and 10 µL of MTT solution were added to each well, and the incubation was kept for 3 h. After this incubation period, the MTT solution was carefully discarded, and 200 µL of DMSO was added to dissolve the formazan crystals. The scaffolds were then incubated for an additional 1 h to ensure complete dissolution. Finally, the media from the wells were taken, and the absorbance values of the solutions were measured via a Dynamic LEDETECT96 microplate reader at 540 nm [60].

### Statistical analysis

All statistical data analyses were conducted using ANOVA with GraphPad Prism version 8 software (GraphPad Software Inc., San Diego, CA, U.S.A). The values are presented as means ± standard deviation (SD), and statistical differences were analyzed by one-way ANOVA and Tukey and Dunnett multiple comparison tests. In all instances, *P* < 0.05 was deemed statistically significant.

## Results

### Raman spectra of GO and rGO

**Fig. 2:**
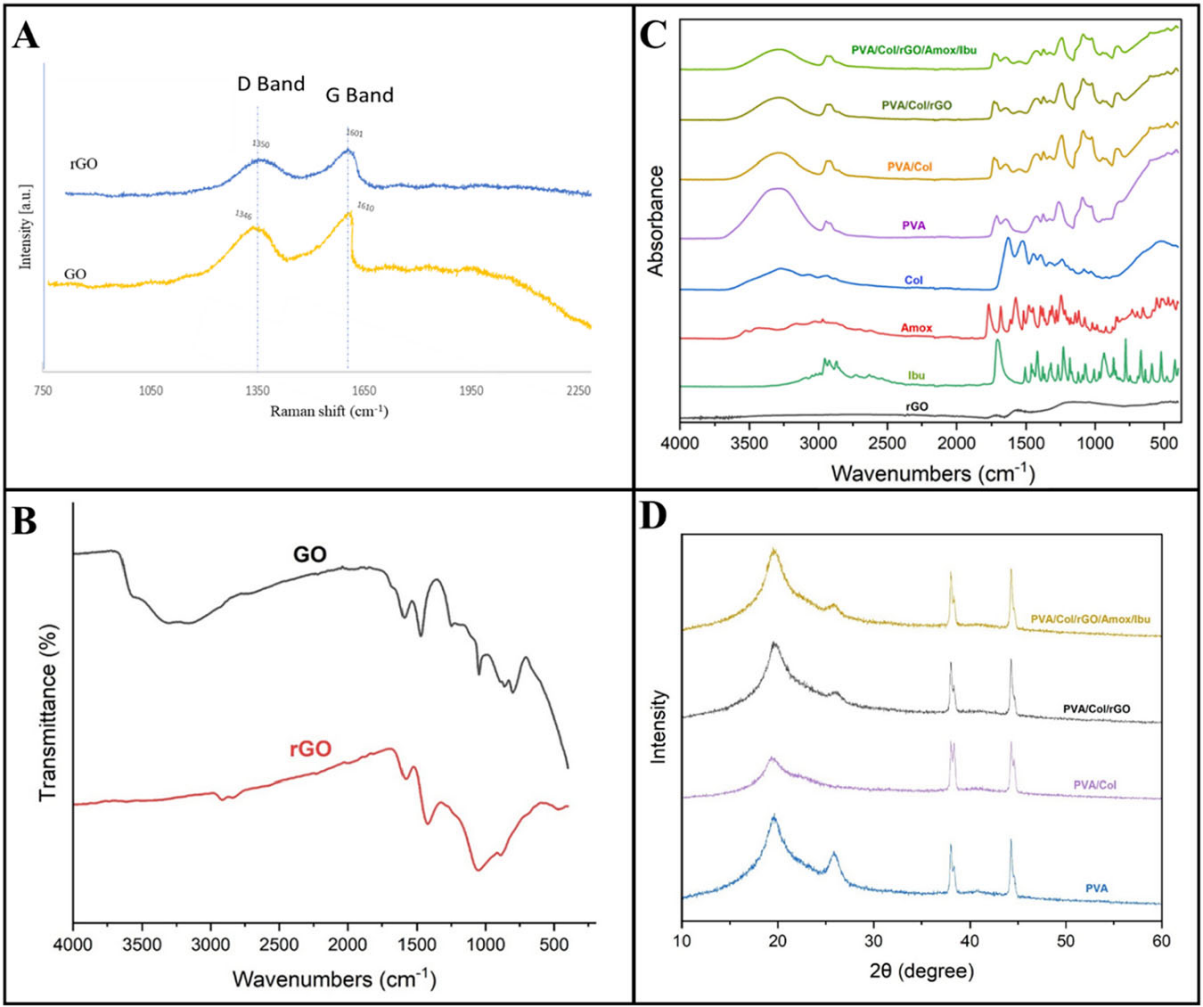
**A** Raman spectra of GO and rGO, **B** FTIR spectra of GO and rGO, **C** FTIR spectra of pure components and 3D biofunctional and conductive neural scaffolds, **D** XRD patterns of the 3D biofunctional and conductive neural scaffolds

Raman spectroscopy is a valuable tool for characterizing carbon-based materials due to its sensitivity to structural changes, particularly in sp²- and sp³-hybridized carbon atoms. As shown in Fig. 2A, both spectra exhibit two characteristic peaks: the D band at approximately 1350 cm⁻¹, associated with disordered sp³-hybridized carbon, and the G band around 1600 cm⁻¹, corresponding to the in-plane vibration of sp²-hybridized carbon atoms. For GO, the D and G bands were observed at 1350 cm⁻¹ and 1610 cm⁻¹, respectively. In contrast, rGO exhibited these bands at 1346 cm⁻¹ (D band) and 1601 cm⁻¹ (G band). The intensity ratio of the D to G bands (I_D/I_G) was calculated to be 0.8 for GO. Following reduction, this ratio decreased to 0.7 for rGO, indicating a change in the carbon structure after the reduction process [61].

### FTIR spectra of the 3D biofunctional and conductive neural scaffolds

The FTIR spectra of GO and rGO are presented in Fig. 2B. The GO spectrum exhibits several characteristic absorption bands corresponding to oxygen-containing functional groups. A broad and intense peak at approximately 3338 cm⁻¹ is attributed to O–H stretching vibrations, indicating the presence of hydroxyl groups. The strong absorption band observed near 1716 cm⁻¹ corresponds to the C=O stretching vibration of carbonyl or carboxylic acid groups. Additionally, the band at 1635 cm⁻¹ is assigned to C=C skeletal vibrations of the aromatic structure. Peaks located at 1154 cm⁻¹ and 1033 cm⁻¹ are associated with C–OH and C–O stretching vibrations, respectively, suggesting the presence of epoxy or alkoxy functional groups. In contrast, the FTIR spectrum of rGO shows notable differences. The broad O–H stretching peak at 3338 cm⁻¹ is no longer observed. The intensity of the C=O stretching peak around 1716 cm⁻¹ is significantly reduced. Moreover, the C=C vibration band shifts from 1635 cm⁻¹ to approximately 1586 cm⁻¹. A residual peak around 1164 cm⁻¹ remains detectable in the rGO spectrum [62–64].

FTIR analysis was conducted to evaluate the chemical composition of the developed 3D scaffolds, including PVA/Col, PVA/Col/rGO, and PVA/Col/rGO/Amox/Ibu. As shown in Fig. 2C, characteristic absorption bands corresponding to each individual component, PVA, collagen (Col), rGO, amoxicillin (Amox), and ibuprofen (Ibu) were detected within the composite spectra. PVA exhibited its typical vibrational bands, including broad O–H stretching in the range of 3500–3000 cm⁻¹, C–H stretching near 2900 cm⁻¹, and C–O stretching around 1080 cm⁻¹. Collagen-specific peaks were identified by the presence of Amide A (~3300 cm⁻¹), Amide I (~1625 cm⁻¹), and Amide II (~1530 cm⁻¹), confirming its polypeptide backbone. The rGO component showed characteristic aromatic C=C stretching vibrations around 1600 cm⁻¹ along with a reduced O–H band intensity. Amoxicillin exhibited characteristic –OH and –NH stretching vibrations in the range of 3450–3000 cm⁻¹ and β-lactam/amide C=O stretching bands between 1775 and 1680 cm⁻¹. Ibuprofen showed aliphatic C–H stretching vibrations at 2950–2850 cm⁻¹ and a carboxylic C=O stretching band near 1715 cm⁻¹. The FTIR spectra of the composite scaffolds showed overlapping and slight shifts in several absorption bands compared to the individual components [65–67].

### XRD patterns of the 3D biofunctional and conductive neural scaffolds

XRD analysis was performed to investigate the crystallinity and structural features of the developed 3D scaffolds (PVA/Col, PVA/Col/rGO, and PVA/Col/rGO/Amox/Ibu), as shown in Fig. 2D. The pure PVA sample exhibited three distinct diffraction peaks at approximately 2θ ≈ 19.5°, 25°, and 40.5°, indicating its semi-crystalline structure. In the PVA/Col scaffold, a broad diffraction peak centered around 2θ ≈ 19.5° was observed. Compared to pure PVA, the characteristic peaks appeared broadened and less intense. The XRD pattern of the PVA/Col/rGO scaffold revealed additional structural features, including the emergence of a low-intensity diffraction peak at approximately 2θ ≈ 26.5°. Furthermore, diffraction peaks in the higher-angle region (~37–39°) appeared more pronounced relative to the PVA/Col scaffold. For the PVA/Col/rGO/Amox/Ibu scaffold, the diffraction pattern was dominated by a broad and low-intensity peak at approximately 2θ ≈ 19.5°, with no distinct crystalline peaks attributable to the drug components [65, 66, 68, 69].

### Four-point prob method

The electrical conductivity values of dry PVA/Col/rGO scaffolds prepared with different rGO concentrations (0.25%, 0.5%, 1%, and 1.5% wt% with respect to total polymer content) are presented in Table 3. Scaffolds containing 0.25% and 0.5% rGO exhibited very low electrical conductivity. In contrast, scaffolds with 1% and 1.5% rGO showed markedly higher conductivity values. No substantial difference was observed between the conductivity values of the 1% and 1.5% rGO-containing scaffolds. Electrical conductivity measurements of the developed 3D scaffolds (PVA/Col, PVA/Col/rGO, and PVA/Col/rGO/Amox/Ibu) in the dry state, determined using the four-point probe method, are summarized in Table 4 [56]. The electrical conductivity of synthesized pure rGO was measured as 1.57 ± 0.172 S/m. The PVA/Col scaffold exhibited very low conductivity. Incorporation of rGO resulted in a significant increase in conductivity, with the PVA/Col/rGO scaffold showing approximately an order-of-magnitude enhancement. Following the incorporation of amoxicillin and ibuprofen, a slight reduction in electrical conductivity was observed in the PVA/Col/rGO/Amox/Ibu scaffold.

**Table 3:** The different rGO concentrations used in PVA/Col/rGO scaffolds

rGO concentrations	Resistivity (ohm*cm)	Electrical conductivity (S/m)
0.25%	8.59 × 10^5^ ± 3.48 × 10^4^	1.28 × 10^−4^ ± 3.43 × 10^−6^
0.5%	1.61 × 10^5^ ± 2.24 × 10^4^	6.29 × 10^−4^ ± 7.53 × 10^−7^
1%	1.51 × 10^4^ ± 1.94 × 10^3^	6.66 × 10^−3^ ± 8.64 × 10^−4^
1.5%	1.26 × 10^4^ ± 1.16 × 10^3^	7.96 × 10^−3^ ± 6.15 × 10^−4^

Data are presented as mean ± standard deviation (*n* = 3)

**Table 4:** Electrical conductivity of the 3D biofunctional and conductive neural scaffolds

3D Scaffolds	Resistivity (ohm*cm)	Electrical conductivity (S/m)
rGO	6.63 × 10 ± 7.11	1.57 ± 0.172
PVA/Col	1.74 × 10^7^ ± 2.64 × 10^6^	5.88 × 10^−6^ ± 8.68 × 10^−7^
PVA/Col/rGO	1.51 × 10^4^ ± 1.94 × 10^3^	6.66 × 10^−3^ ± 8.64 × 10^−4^
PVA/Col/rGO/Amox/Ibu	1.72 × 10^4^ ± 1.88 × 10^3^	5.83 × 10^−3^ ± 6.48 × 10^−4^

Data are presented as mean ± standard deviation (*n* = 9)

### TEM images of GO and rGO

**Fig. 3:**
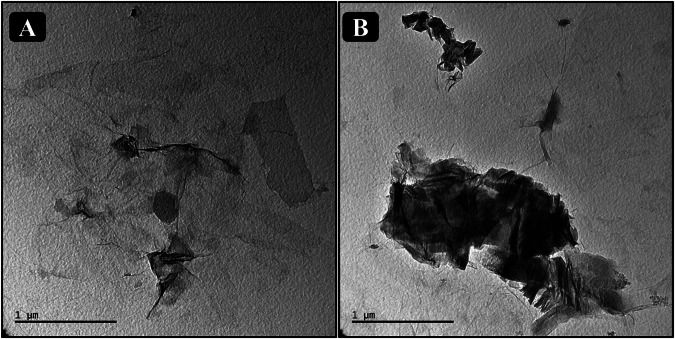
TEM images of (**A**). GO, (**B**) rGO

The morphologies and microstructures of graphene oxide (GO) and reduced graphene oxide (rGO) were examined using transmission electron microscopy (TEM), as shown in Fig. 3. TEM images of GO (Fig. 3A) revealed thin, transparent, and sheet-like structures with slight wrinkles and smooth surfaces. The GO sheets exhibited lateral dimensions in the micron scale, with limited overlapping regions and well-defined edges, indicating a high degree of exfoliation and dispersion. In contrast, TEM images of rGO (Fig. 3B) displayed a crumpled and folded nanosheet morphology with darker contrast. The rGO sheets appeared more compact, showing increased stacking and multilayer aggregation compared to GO. Nanoscale wrinkles and folded regions were clearly visible in the rGO structure.

### Printability calculation based on strand and pore geometry of the 3D biofunctional and conductive neural scaffolds

The printability of the developed 3D scaffolds (PVA, PVA/Col, PVA/Col/rGO, and PVA/Col/rGO/Amox/Ibu) was evaluated based on strand and pore fidelity using optical imaging and quantitative analysis, as summarized in Table 5. All scaffolds were printed with standard dimensions of 30 × 30 mm. A printability value of 1 ± 0.1 was considered acceptable, indicating close agreement between the designed and experimentally fabricated structures. Pure PVA scaffolds exhibited strand and pore printability values of 1.000 and 1.008, respectively. The PVA/Col scaffold showed a strand printability of 0.967 and a pore printability of 1.031. Incorporation of rGO resulted in strand and pore printability values of 0.967 and 0.998, respectively. The PVA/Col/rGO/Amox/Ibu scaffold exhibited a strand printability of 0.933 and a pore printability of 1.005 [57].

**Table 5:** Optical characterization and printability performance of the 3D biofunctional and conductive neural scaffolds

Solution composition	3D-printed scaffolds	Optical microscope images	Strand printability	Pore printability
PVA			1	1.008
PVA/Col			0.9667	1.031
PVA/Col/rGO			0.9667	0.998
PVA/Col/rGO/Amox/Ibu			0.9333	1.005

### SEM images of the 3D biofunctional and conductive neural scaffolds

**Fig. 4:**
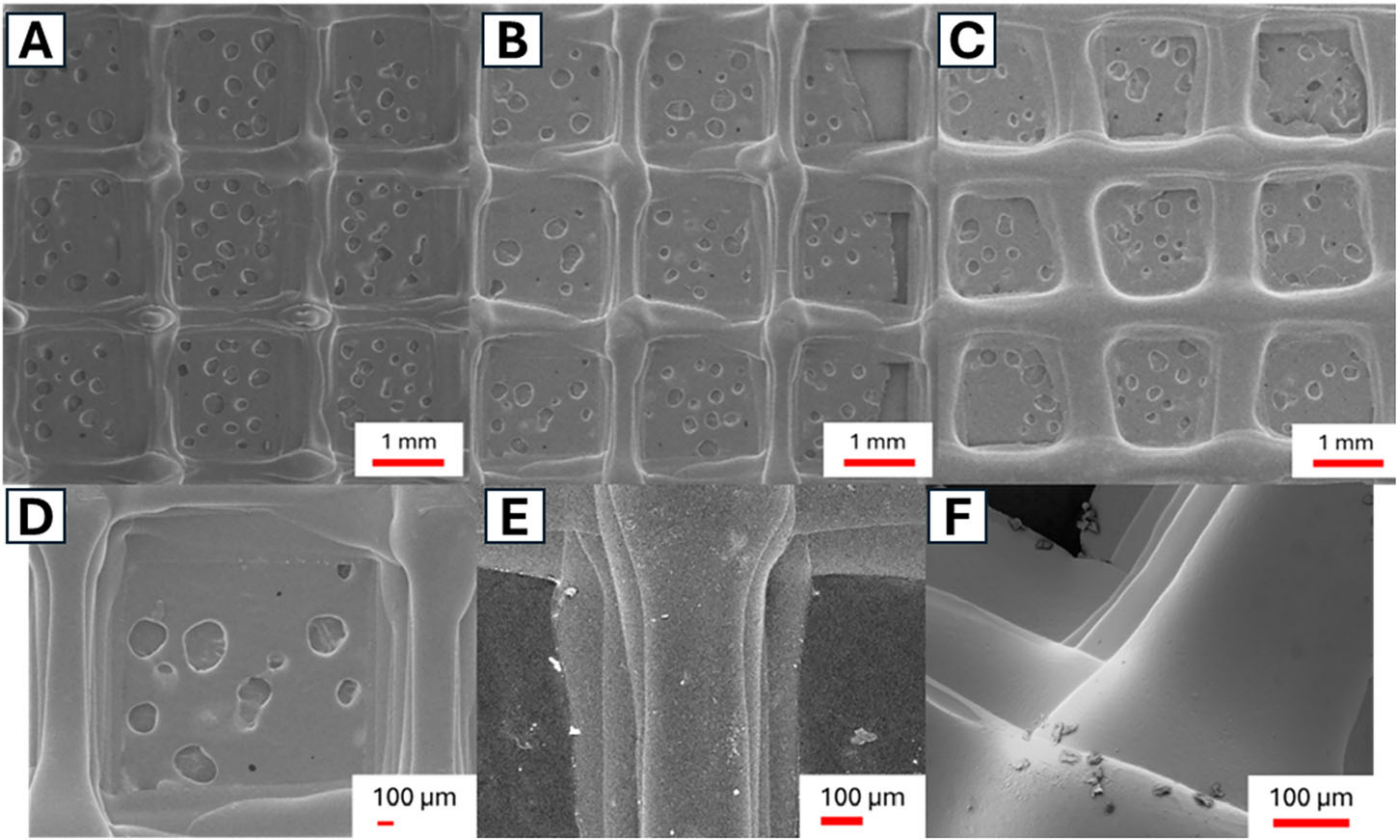
SEM images of the 3D Biofunctional and Conductive Neural Scaffolds. **A**, **D** PVA/Col; **B**, **E** PVA/Col/rGO; **C**, **F** PVA/Col/rGO/Amox/Ibu

The microstructural characteristics of the developed 3D scaffolds were examined using SEM. In some SEM images, small pores observed at the base of the scaffolds were attributed to the double-sided carbon tape used for sample mounting during imaging. SEM images of the PVA/Col scaffold (Fig. 4A, D) revealed a structure composed of well-defined, regularly repeating square-shaped macropores. The filament layers were deposited in an ordered pattern; however, slight variations in layer height were observed in certain regions. The PVA/Col/rGO scaffold (Fig. 4B, E) exhibited a more clearly defined layered architecture. Higher-magnification images (Fig. 4E) showed sequential stacking of filament strands with continuous interlayer contact. The surface of the filaments appeared smoother and more compact compared to the PVA/Col scaffold. The PVA/Col/rGO/Amox/Ibu scaffold (Fig. 4C, F) displayed well-aligned layers and continuous filament junctions throughout the structure. The magnified image (Fig. 4F) showed no visible gaps or delamination at the filament interfaces. A distinct layered structure was evident in all scaffold formulations.

### DSC-TGA results of the 3D biofunctional and conductive neural scaffolds

**Fig. 5:**
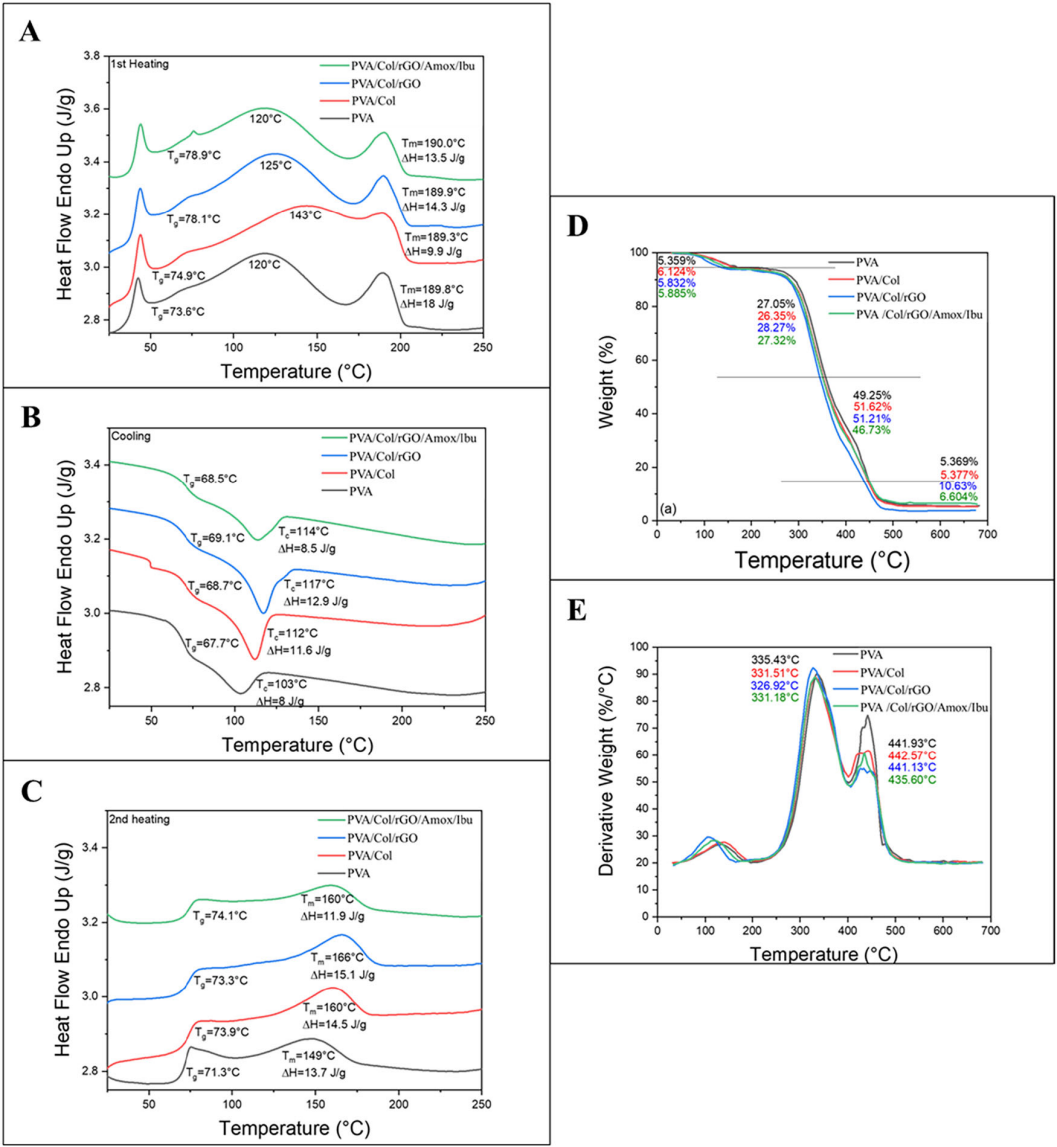
DSC curves during the first heating (**A**), cooling (**B**), and second heating (**C**) of the 3D biofunctional and conductive neural scaffolds. **D** TGA and **E** DTG curves of the 3D biofunctional and conductive neural scaffolds

The thermal properties of the developed 3D scaffolds (PVA, PVA/Col, PVA/Col/rGO, and PVA/Col/rGO/Amox/Ibu) were evaluated using differential scanning calorimetry (DSC) and thermogravimetric analysis (TGA), and derivative thermogravimetry (DTG), as shown in Fig. 5. DSC analysis provided glass transition temperature (Tg), melting temperature (Tm), and enthalpy change (ΔH) values, while TGA and DTG analyses revealed degradation behavior and thermal stability. Figure 5A–C presents the DSC thermograms during the first heating, cooling, and second heating cycles, respectively. Pure PVA exhibited a Tg of 73.6 °C, a Tm of 189.8 °C, and a ΔH of 18 J/g. The PVA/Col scaffold showed an increased Tg of 74.9 °C and a reduced ΔH of 9.9 J/g. Upon incorporation of rGO, Tg increased to 78.1 °C and ΔH increased to 14.3 J/g. The PVA/Col/rGO/Amox/Ibu scaffold exhibited the highest Tg value (78.9 °C), a Tm of 190.0 °C, and a ΔH of 13.5 J/g. TGA analysis showed an initial weight loss between 100 and 150 °C for all scaffolds. All samples exhibited an initial weight loss of approximately 5–6% below 100 °C, attributed to moisture and residual solvent evaporation. The highest initial weight loss was observed in the PVA/Col scaffold (6.124%). The main thermal degradation occurred between 250 and 450 °C, with total mass losses of 51.62% for PVA/Col and 46.73% for PVA/Col/rGO/Amox/Ibu. The thermal stability and degradation kinetics are captured in the TGA (Fig. 5D) and DTG (Fig. 5E) curves. DTG curves revealed shifts in peak decomposition temperatures depending on scaffold composition.

### Mechanical properties of the 3D biofunctional and conductive neural scaffolds

**Fig. 6:**
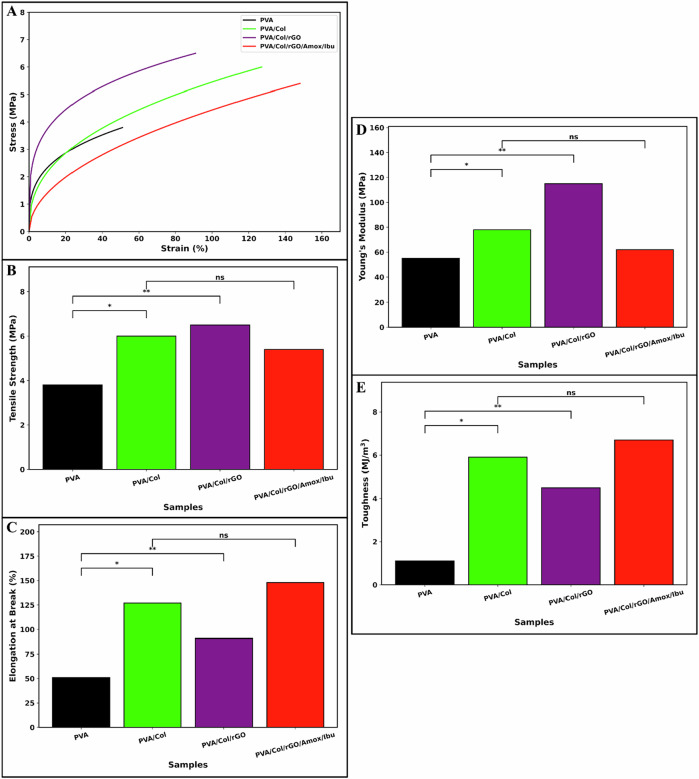
Mechanical properties of the 3D biofunctional and conductive neural scaffolds. **A** Representative engineering stress–strain curves, **B** ultimate tensile strength (UTS), **C** elongation at break, **D** Young’s modulus, and **E** tensile toughness

The mechanical performance of the developed 3D scaffolds (PVA, PVA/Col, PVA/Col/rGO, and PVA/Col/rGO/Amox/Ibu) was rigorously analyzed to evaluate their structural integrity and deformation behavior under uniaxial tensile loading. Representative stress–strain curves comparing the four formulations are shown in Fig. 6A. These curves illustrate the elastic and plastic deformation behavior, maximum tensile stress, and elongation at break of each scaffold. The pristine PVA scaffold exhibited a tensile strength of 3.8 MPa and a failure strain of 51%, indicating limited ductility. The incorporation of collagen (PVA/Col) improved both tensile strength and elongation at break compared with pure PVA. The addition of rGO (PVA/Col/rGO) resulted in the highest tensile strength, reaching 6.5 MPa. In contrast, the drug-loaded scaffold (PVA/Col/rGO/Amox/Ibu) showed a slightly reduced peak stress of 5.4 MPa, but a markedly increased elongation at break of approximately 150%. Statistical comparisons of the mechanical parameters are summarized in Fig. 6B–E. The Ultimate Tensile Strength (UTS) values shown in Fig. 6B demonstrate a statistically significant increase (*p* < 0.01) for all modified scaffolds compared with the PVA control. Figure 6C presents the elongation at break, where the PVA/Col/rGO/Amox/Ibu scaffold exhibited the highest strain values, significantly higher (*p* < 0.01) than the control group. The elastic properties and energy absorption capacities were further quantified through the Young’s Modulus and Toughness. The Young’s modulus, calculated from the linear elastic region of the stress–strain curves, is shown in Fig. 6D. The rGO-reinforced scaffold exhibited the highest modulus of 115 MPa. The tensile toughness, calculated from the area under the stress–strain curve, is presented in Fig. 6E. The PVA/Col/rGO/Amox/Ibu scaffold showed the highest toughness value of approximately 6.7 MJ/m³, representing a statistically significant improvement (*p* < 0.01) compared with the control.

### In vitro degradation of the 3D biofunctional and conductive neural scaffolds

**Fig. 7:**
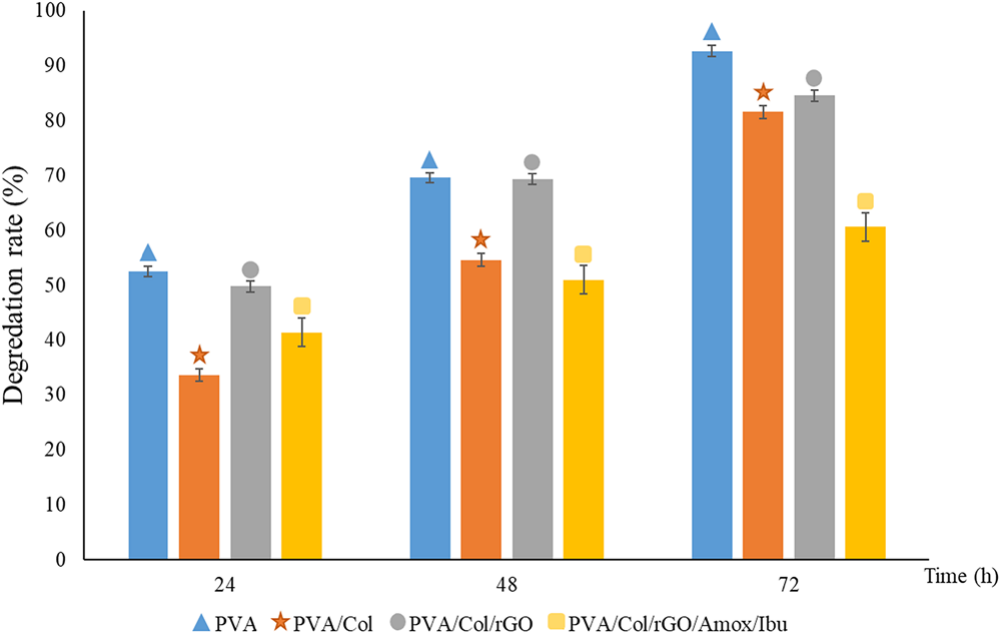
Degradation rate of the 3D biofunctional and conductive neural scaffolds, *mean* *±* *SD values of n* = *3 independent experiments*

The degradation behavior of the developed 3D scaffolds (PVA, PVA/Col, PVA/Col/rGO, and PVA/Col/rGO/Amox/Ibu) was evaluated by measuring weight loss after immersion in phosphate-buffered saline (PBS, pH 7.4) at 37 °C for up to 72 h, as shown in Fig. 7. Weight loss percentage was used to quantify degradation rates. Pure PVA exhibited the highest degradation rate, reaching approximately 90% weight loss by day 3. The PVA/Col scaffold showed a reduced degradation rate compared to pure PVA, with approximately 80% weight loss observed within 72 h. The PVA/Col/rGO scaffold exhibited further stabilization, with weight loss limited to approximately 85% at day 3. Among all formulations, the PVA/Col/rGO/Amox/Ibu scaffold demonstrated the lowest degradation rate, showing weight loss slightly above 60% after 72 h.

### Evaluation of controlled drug release from the 3D biofunctional and conductive neural scaffolds

**Fig. 8:**
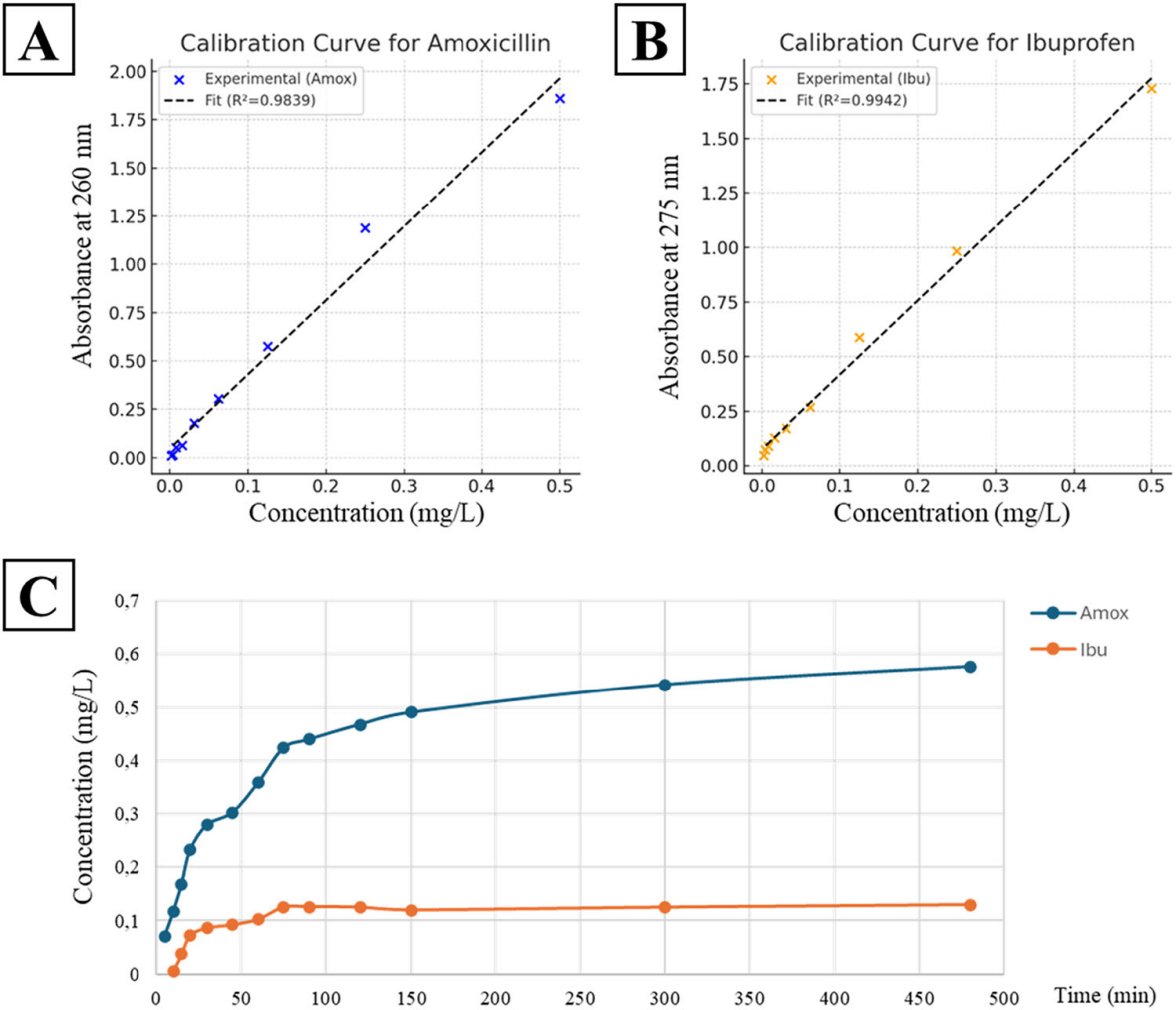
Calibration curve for Amox (**A**) and Ibu (**B**). conductive neural scaffold (PVA/Col/rGO/Amox/Ibu). Cumulative drug release profiles of Amox and Ibu from the 3D biofunctional and (**C**)

The cumulative release behavior of amoxicillin (Amox) and ibuprofen (Ibu) from the 3D biofunctional and conductive neural scaffolds (PVA/Col/rGO/Amox/Ibu) was evaluated using a UV–Vis spectrophotometer. Drug concentrations were quantified by measuring absorbance at 260 nm for Amox and 275 nm for Ibu, and calibration curves for both drugs are presented in Fig. 8A, B [70]. The cumulative release profiles of Amox and Ibu are shown in Fig. 8C. Both drugs exhibited an initial rapid release phase within the first 100 min. Amox reached a cumulative concentration of approximately 0.6 mg/L after 480 min, whereas Ibu exhibited a lower cumulative release, reaching a plateau at approximately 0.12 mg/L over the same period.

### Modeling and analysis of drug release kinetics of the 3D biofunctional and conductive neural scaffolds

**Fig. 9:**
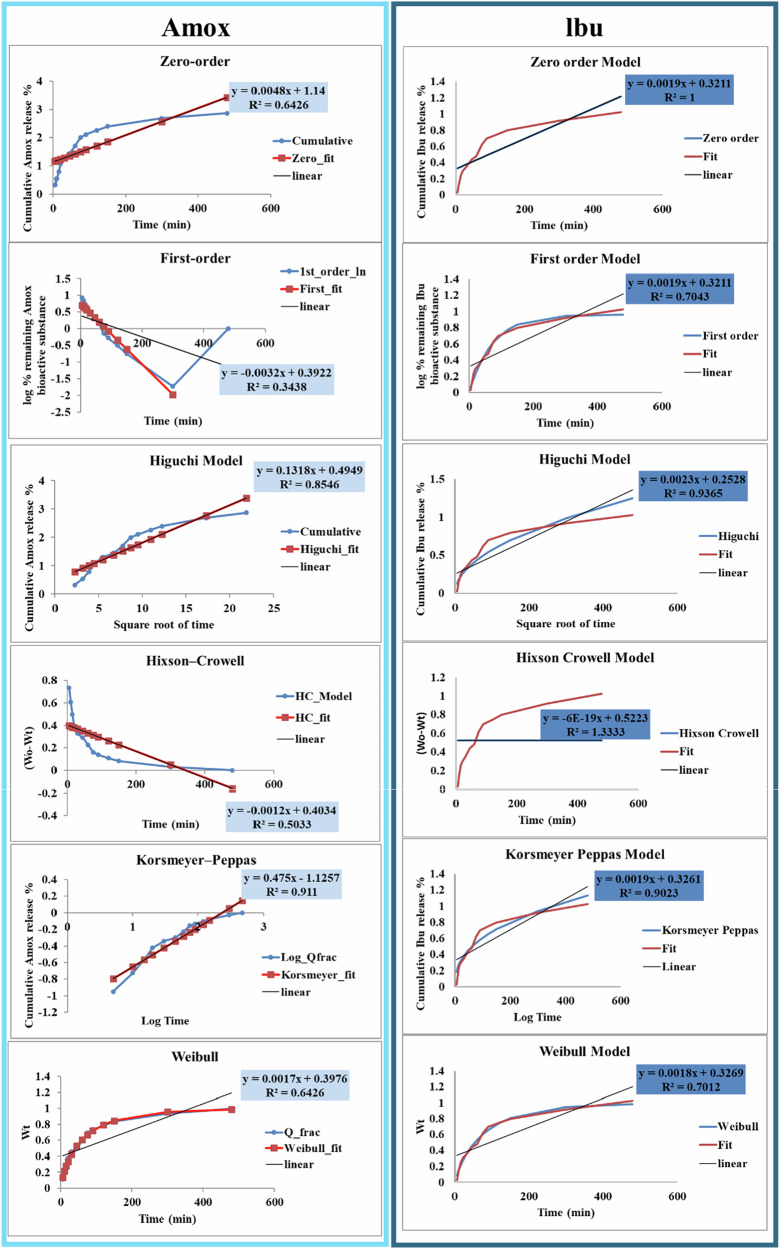
Amox and Ibu release kinetics models of the 3D biofunctional and conductive neural scaffold

The release kinetics of Amox and Ibu from the PVA/Col/rGO/Amox/Ibu scaffold were evaluated using six kinetic models: Zero-order, First-order, Higuchi, Hixson–Crowell, Korsmeyer–Peppas, and Weibull models (Table 6, Fig. 9). For the combined drug system, the Zero-order model showed a moderate fit, whereas the First-order model exhibited lower *R*² values compared to other models. The Higuchi model demonstrated a strong correlation with the experimental data (*R*² > 0.9), indicating diffusion-dominated release behavior. The Hixson–Crowell model also showed a reasonable fit, suggesting the contribution of matrix erosion. The Korsmeyer–Peppas model yielded an *n* value corresponding to anomalous (non-Fickian) transport. Among all evaluated models, the Weibull model provided the best fit with *R*² values exceeding 0.97, indicating a complex, time-dependent release profile. Drug-specific kinetic analysis revealed distinct release behaviors. For Amox, the highest correlation was obtained with the Korsmeyer–Peppas model (*R²* = 0.911), followed by the Higuchi model (*R²* = 0.8546). In contrast, the First-order (*R²* = 0.3438) and Hixson–Crowell (*R*² = 0.5033) models showed poor correlation. For Ibu, the Higuchi model exhibited the highest *R*² value (0.9365), followed by the Korsmeyer–Peppas model (*R*² = 0.9023). The First-order model showed a moderate fit (*R²* = 0.7043). The Hixson–Crowell model yielded an anomalously high *R*² value (1.3333), suggesting limited applicability. The Weibull model showed moderate correlations for both Amox (*R²* = 0.6426) and Ibu (*R²* = 0.7012).

**Table 6:** Comparison of the equation form, regression parameters, and *R*² values of each model

Release of	Kinetic models	Equations	Coefficients, Buffer pH 7.4		
			*K (K*_*o*_*, K*_*1*_*, K*_*H*_ *and K*_*r*_*)*	*R*²	*n*
Amox	**Zero-order**	*C*_*t*_ = *C*_*0*_ + *K*_*0*_*t*	K₀ = 0.0047, C₀ = 1.139	0.6426	
	**First-order**	log Q_1_ = log Q_0_ + K_1_t/2.303	K = −0.0090, Q_0_ = 0.749	0.9553	
	**Higuchi**	*f*_t_ = Q = K_H_ t^1/2^	K_H_ = 0.1318	0.8546	
	**Hixson–Crowell**	(W₀)^(1/3) − (W_t_)^(1/3) = k·t	W₀ = −0.0011, *k* = 0.40	0.5033	
	**Korsmeyer–Peppas**	Mt/M ∞ = K_r_t^n^ + *b*	K = 0.4750, *n* = 1.1256	0.911	≈0.475
	**Weibull (non-linear)**	F = 1 − exp[−(t/α)^β]	α = 65.1237, β = 0.7520	0.9922	
Ibu	**Zero-order**	*C*_*t*_ = *C*_*0*_ + *K*_*0*_*t*	K₀ = 0.0019, C₀ = 0.3211	0.7043	–
	**First-order**	log Q_1_ = log Q_0_ + K_1_t/2.303	K = 0.8124, Q_0_ = 0.9641	0.9798	–
	**Higuchi**	*f*_t_ = Q = K_H_ t^1/2^	K_H_ = 0.0569	0.8761	–
	**Hixson–Crowell**	(W₀)^(1/3) − (W_t_)^(1/3) = k·t	W₀ = 0.5223, *k* = 0.0000	0.0	–
	**Korsmeyer–Peppas**	Mt/M ∞ = K_r_t^n^ + *b*	K = 0.0977, n = 0.3968	0.9251	0.3968
	**Weibull (non-linear)**	F = 1 − exp[−(t/α)^β]	α = 82.6389, β = 0.8420	0.9876	–

### Antibacterial performance of the 3D biofunctional and conductive neural scaffolds

**Fig. 10:**
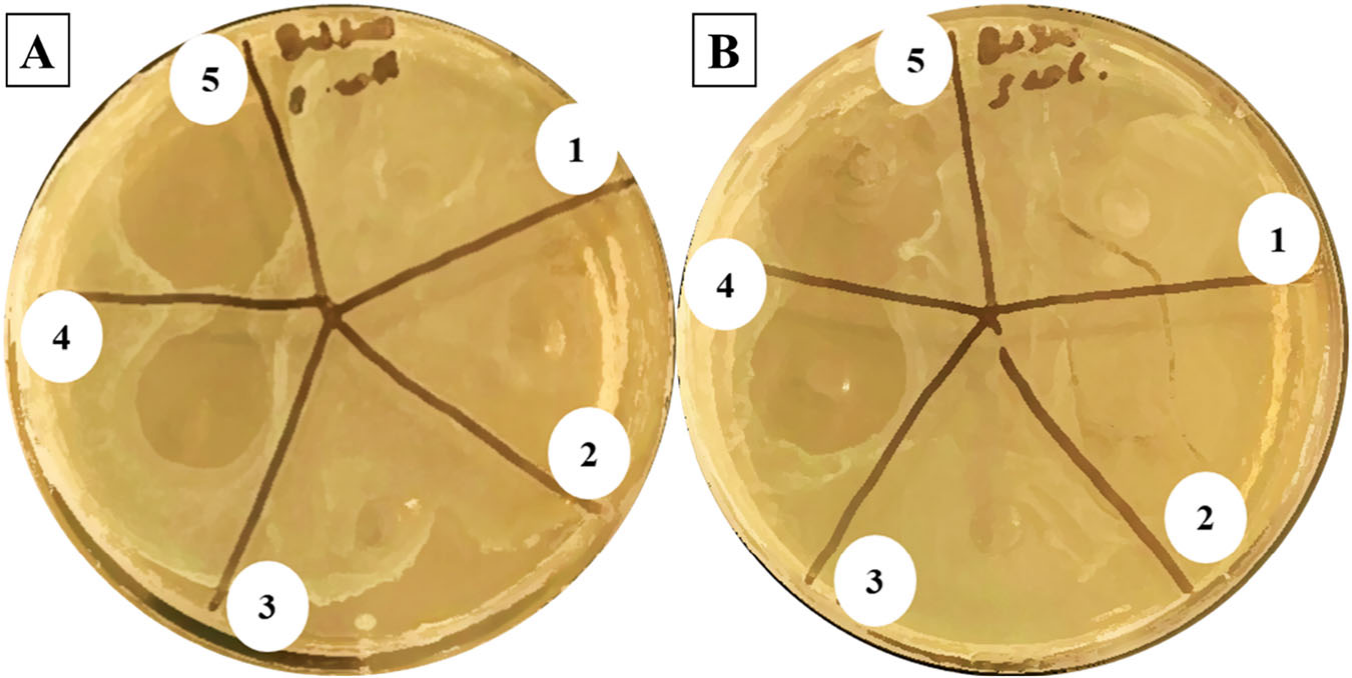
Antibacterial activity of the 3D biofunctional and conductive neural scaffolds against **A** *E.coli* and **B** *S.aureus* evaluated by the disk diffusion method. (1) PVA/Col, (2) PVA/Col/rGO, (3) PVA/Col/rGO/Ibu, (4) PVA/Col/rGO/Amox, (5) PVA/Col/rGO/Amox/Ibu

The antibacterial activity of the 3D biofunctional and conductive neural scaffolds PVA/Col (1), PVA/Col/rGO (2), PVA/Col/rGO/Ibu (3), PVA/Col/rGO/Amox (4), and PVA/Col/rGO/Amox/Ibu (5) was evaluated against *Escherichia coli* and *Staphylococcus aureus* using the disk diffusion method. As shown in Fig. 10, the scaffolds were placed on agar plates inoculated with bacterial cultures, and inhibition zone diameters were measured after 24 h of incubation. For *E. coli* (Fig. 10A), inhibition zones of 16.84 ± 2.06 mm and 28.30 ± 3.32 mm were observed for the PVA/Col/rGO/Amox and PVA/Col/rGO/Amox/Ibu scaffolds, respectively. Similarly, for *S. aureus* (Fig. 10B), inhibition zone diameters of 18.94 ± 2.81 mm and 18.34 ± 2.83 mm were recorded for the same formulations. In contrast, scaffolds without amoxicillin (PVA/Col, PVA/Col/rGO, and PVA/Col/rGO/Ibu) exhibited negligible or no inhibition zones against either bacterial strain, indicating minimal antibacterial activity in these groups.

The inhibition zones were observed in scaffolds containing amoxicillin (4 and 5), demonstrating effective antibacterial properties against both gram-negative and gram-positive bacteria.

### Cytocompatibility of the 3D biofunctional and conductive neural scaffolds

**Fig. 11:**
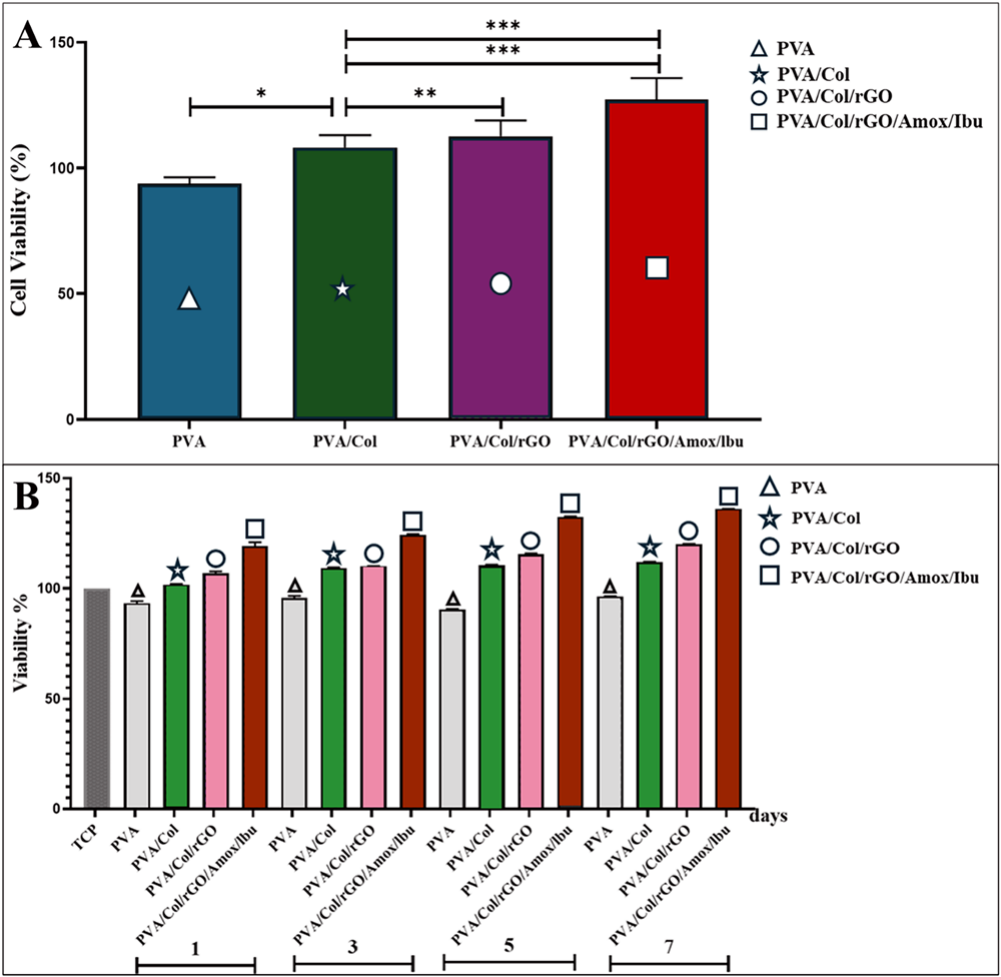
**A** Cell viability results are given as % of the negative control, *mean* *±* *SD values of n* = *3 independent experiments*. ^***^P < 0.001, and ^****^P < 0.0001 vs. negative control group. *Data was analyzed using one-way analysis of variance (ANOVA) and the Tukey test*. **B** Cell viability of L929 cells exposed to different concentrations (days 1, 3, 5, and 7) of the 3D biofunctional and conductive neural scaffolds (PVA, PVA/Col, PVA/Col/rGO, PVA/Col/rGO/Amox/Ibu). *Data represents standard deviations (n* = *3)*, ^*^P < 0 05, ^**^P < 0.01, and ^***^P < 0.001 vs. control group; ^#^ *P* < 0.05, and ^##^*P* < 0.01 vs. the developed 3D scaffolds. *Data was analyzed using one-way analysis of variance (ANOVA) and the Dunnett test*

In this study, the effects of 3D biofunctional and conductive neural scaffolds containing different compositions (PVA, PVA/Col, PVA/Col/rGO, and PVA/Col/rGO/Amox/Ibu) on cell viability were evaluated to assess their biocompatibility. As shown in Fig. 11A, scaffolds containing collagen, rGO, and the dual-drug system exhibited significantly enhanced cell viability compared to the pure PVA scaffold. Among all groups, the PVA/Col/rGO/Amox/Ibu scaffold demonstrated the highest cell viability, with statistically significant increases compared to PVA (*p* < 0.05), PVA/Col (*p < 0.01), and PVA/Col/rGO (**p < 0.001). The incorporation of collagen promoted cell adhesion and proliferation, while the addition of rGO further improved the biological response by enhancing cell–material interactions. Time-dependent cytotoxicity analysis was performed on days 1, 3, 5, and 7 (Fig. 11B). The PVA scaffold showed moderate initial cell viability, whereas PVA/Col and PVA/Col/rGO scaffolds exhibited progressively higher proliferation rates over time. Notably, the PVA/Col/rGO/Amox/Ibu group maintained the highest cell viability throughout the experimental period. By day 7, cell viability in this group was comparable to or higher than that of the TCP (tissue culture plate) control.

## Discussion

### Raman spectroscopy analysis of GO and rGO

The presence of prominent D and G bands in both GO and rGO confirms the carbon-based structure and is consistent with previously reported Raman signatures of graphene-derived materials (Fig. 2A). The relatively high I_D/I_G ratio observed in GO reflects the significant structural disorder introduced by oxygen-containing functional groups during oxidation. After reduction, the decrease in the I_D/I_G ratio suggests partial restoration of sp² carbon domains and an improvement in structural order, which is commonly associated with the removal of oxygen functionalities. Similar trends have been reported in the literature, where chemical or thermal reduction of GO leads to reorganization of the graphene lattice rather than complete defect elimination [71, 72]. However, the persistence of the D band in rGO indicates that some structural defects remain, highlighting that the reduction process does not fully recover a pristine graphene structure. This behavior aligns with previously published studies and supports the effectiveness, yet inherent limitations, of GO reduction methods [44].

### FTIR analysis of the 3D biofunctional and conductive neural scaffolds

The FTIR results clearly demonstrate the successful reduction of GO to rGO through the substantial removal of oxygen-containing functional groups (Fig. 2B). The disappearance of the O–H stretching band and the marked decrease in the C=O peak intensity indicate effective elimination of hydroxyl and carbonyl groups during the reduction process, which is consistent with previously reported studies. The shift of the C=C vibration band toward lower wavenumbers suggests partial restoration of the conjugated sp² carbon network, a key feature associated with graphene-like structures. However, the presence of a residual C–OH-related peak implies that the reduction process does not fully remove all oxygen functionalities, which is a commonly reported limitation of thermal reduction methods. Similar observations have been described in the literature, where incomplete reduction leads to a balance between improved conductivity and retained surface functionality [73, 74]. This partial reduction can be advantageous for certain electronic and biomedical applications, as it enhances electrical conductivity while preserving sufficient surface chemistry for functionalization and interfacial interactions.

The FTIR results confirm the successful incorporation of PVA, collagen, rGO, and the therapeutic agents amoxicillin and ibuprofen within the 3D scaffold matrix (Fig. 2C). The presence of characteristic functional group vibrations from each component indicates that the fabrication process preserved their chemical integrity, consistent with previous reports on multicomponent polymeric scaffolds. The observed band merging and slight peak shifts in the composite scaffolds suggest the presence of physical interactions, predominantly hydrogen bonding, among the polymer chains, rGO sheets, and drug molecules. Similar spectral behavior has been reported in literature for PVA–collagen systems and graphene-based nanocomposites, where non-covalent interactions dominate the composite structure [25, 71]. Although distinct drug-related peaks were not clearly resolved in the final scaffold spectra, likely due to their relatively low concentrations and overlap with polymer bands, the overall spectral changes support the successful integration of both drugs into the scaffold matrix. This interaction-driven incorporation is particularly relevant for controlled drug delivery applications, where physical entrapment and intermolecular bonding play a critical role in sustained release behavior [75–77].

### XRD analysis of the 3D biofunctional and conductive neural scaffolds

The XRD results demonstrate that the crystallinity of the scaffolds is strongly influenced by the incorporation of collagen, rGO, and drug molecules (Fig. 2D). The characteristic diffraction peaks observed in pure PVA are consistent with its semi-crystalline nature, arising from intermolecular hydrogen bonding and partial chain ordering, as widely reported for PVA-based systems [78]. The broadening and attenuation of these peaks upon collagen addition indicate disruption of the PVA crystalline domains by the amorphous collagen structure, in agreement with previous studies describing collagen’s weak and diffuse diffraction behavior [79]. The appearance of a diffraction peak at approximately 2θ ≈ 26.5° following rGO incorporation confirms the successful reduction of GO and its presence within the polymer matrix [80]. Additionally, the sharpening of higher-angle reflections suggests localized structural reordering, which may be attributed to π–π interactions between rGO nanosheets and polymer chains, promoting partial stacking and improved molecular alignment [80]. In contrast, the further reduction in peak intensity and increased peak broadening observed in the PVA/Col/rGO/Amox/Ibu scaffold indicate a decrease in overall crystallinity. This behavior is consistent with the amorphous nature of amoxicillin and ibuprofen, which can interfere with polymer chain packing and disrupt polymer–filler interactions [59]. Overall, these findings suggest that while collagen and drug incorporation increase the amorphous character of the scaffold, rGO plays a compensatory role by inducing partial structural reorganization.

### Evaluation of electrical conductivity via the four-point probe method

The conductivity results demonstrate that rGO concentration plays a critical role in establishing electrically conductive pathways within the PVA/Col scaffold matrix. The low conductivity observed at 0.25% and 0.5% rGO suggests that the rGO content is below the percolation threshold required for the formation of continuous conductive networks. The significant increase in conductivity at 1% rGO indicates successful network formation, while the absence of a meaningful conductivity gain at 1.5% (Table 3). Considering the comparable conductivity values and the potential cytotoxic effects associated with higher rGO contents, 1% rGO was selected as the optimal concentration for subsequent scaffold formulations [81, 82] (Table 4). The inherently insulating nature of PVA and collagen explains the low conductivity of the PVA/Col scaffold, whereas the substantial conductivity enhancement upon rGO incorporation confirms its effectiveness in facilitating charge transport [83]. Similar conductivity improvements have been reported in graphene-based polymer composites, where rGO acts as a conductive filler enabling electron transport through interconnected nanosheets [84]. The slight decrease in conductivity after drug incorporation can be attributed to partial disruption of the conductive network by amorphous drug molecules occupying interfacial regions within the scaffold. The electrical conductivity of the developed PVA/Col/rGO/Amox/Ibu scaffold (5.83 × 10^−3^ S/m) was quantitatively compared with native peripheral nerve tissue and previously reported conductive neural scaffolds. Native peripheral nerve conductivity has been reported in the range of 0.02–0.6 S/m depending on anatomical location and measurement conditions [85]. Although the obtained value is lower than the physiological conductivity of intact nerve tissue, it falls within the functional electroactive range (10^−3^–10^−1^ S/m) reported for conductive scaffolds used in neural tissue engineering [86–89]. Previous studies have demonstrated that scaffolds exhibiting conductive behavior within 10^−2^–10^−6^ S/m are sufficient to support neurite extension, Schwann cell communication, and bioelectrical signal transmission [90, 91]. In particular, rGO-modified polymeric scaffolds have shown conductivities between 0.002 and 0.04 S/m [92], while aligned PPy/SF systems have been reported in the range of 0.001–0.113 S/m [93]. Collectively, despite not fully matching the conductivity of native peripheral nerve tissue, these comparisons confirm that the conductivity of the PVA/Col/rGO/Amox/Ibu scaffold resides within the functional electroactive range and is sufficient to facilitate localized electrical signaling and support neural cell communication and neural cell activity [86, 91, 93, 94]. Overall, these findings indicate that the developed scaffold successfully balances electrical functionality with bioactivity, reinforcing its potential applicability in neural tissue engineering throughout the degradation process.

### TEM characterization of GO and rGO

The TEM observations highlight distinct morphological differences between GO and rGO resulting from the reduction process (Fig. 3). The highly transparent and lightly contrasted GO sheets are characteristic of monolayer or few-layer structures enriched with oxygen-containing functional groups, which disrupt the π-conjugated carbon network and promote exfoliation (Fig. 3A). The limited aggregation and smooth sheet morphology observed for GO are consistent with its hydrophilic nature and favorable dispersion behavior, which are advantageous for surface functionalization and uniform incorporation into polymer matrices. Following reduction, the rGO sheets exhibit darker contrast, increased folding, and pronounced stacking, reflecting partial restoration of the sp² carbon network and a reduction in oxygen functionalities [95, 96]. The re-emergence of hydrophobic interactions and van der Waals forces between graphene layers leads to a more compact and crumpled morphology, as commonly reported for chemically or thermally reduced GO. Although this structural rearrangement can reduce dispersion stability, it is often associated with enhanced electrical conductivity and mechanical reinforcement potential. The presence of nanoscale wrinkles and defects in rGO (Fig. 3B) may also increase surface area and provide active sites for drug loading or biomolecular interactions [97]. Collectively, the TEM results confirm the successful structural transition from GO to rGO and demonstrate how reduction-induced morphological changes can be strategically leveraged in composite scaffold formulations, particularly for applications requiring tunable conductivity, mechanical strength, and multifunctional performance.

### Printability analysis based on strand and pore geometry of the 3D biofunctional and conductive neural scaffolds

The printability results demonstrate that all scaffold formulations achieved acceptable structural fidelity within the predefined printability threshold (Table 5). The near-ideal printability observed for pure PVA reflects its favorable rheological behavior and well-established suitability for extrusion-based 3D printing. The slight reduction in strand fidelity following collagen incorporation can be attributed to changes in viscosity and flow behavior introduced by the protein component, while the marginal increase in pore size suggests minor spreading during deposition. The incorporation of rGO appears to enhance pore regularity without compromising strand fidelity, indicating a stabilizing effect on the printed filaments. Similar improvements in print stability have been reported for graphene-based nanocomposite inks, where rGO acts as a rheology modifier and reinforces filament shape retention. In the most complex formulation containing amoxicillin and ibuprofen, the observed decrease in strand printability is likely related to increased viscosity and molecular interactions within the ink. Nevertheless, the pore printability remained within the acceptable range, confirming that drug incorporation does not significantly disrupt the printing process. Overall, these findings confirm the robustness and reproducibility of the printing strategy, demonstrating that high-fidelity 3D scaffolds can be fabricated even with the inclusion of conductive nanomaterials and biofunctional agents [57].

### SEM characterization of the 3D biofunctional and conductive neural scaffolds

The SEM observations confirm that the extrusion-based 3D printing process produced scaffolds with well-defined macroporous architectures across all formulations (Fig. 4). The regular pore geometry observed in the PVA/Col scaffold reflects effective strand deposition; however, the minor variations in layer height suggest limited instability in filament stacking, likely related to the rheological behavior of the PVA/Col ink. The incorporation of rGO led to a more pronounced layered morphology and improved interlayer fusion, indicating enhanced filament stability during printing. Similar effects have been reported in graphene-reinforced polymer inks, where rGO acts as a rheology modifier that optimizes viscosity and promotes shape retention during extrusion [57, 98]. The smoother and denser filament surfaces observed in the PVA/Col/rGO scaffold may be advantageous for cell–material interactions and for maintaining consistent electrical pathways within conductive scaffolds. The PVA/Col/rGO/Amox/Ibu scaffold exhibited the highest degree of structural continuity, with well-aligned layers and uninterrupted filament junctions. Consistent with previous reports, the absence of interfacial voids or delamination is particularly important in ensuring mechanical integrity and long-term stability under physiological conditions [99, 100]. Overall, these results suggest that the combined incorporation of collagen, rGO, and bioactive agents improves layer alignment and surface smoothness, which provides a more favorable microenvironment for neural cell adhesion and proliferation.

### DSC-TGA analysis of the 3D biofunctional and conductive neural scaffolds

The DSC results indicate that scaffold composition significantly influences thermal transitions and molecular organization (Fig. 5). The thermal behavior of pure PVA is consistent with its semi-crystalline nature and strong intermolecular hydrogen bonding. The increase in Tg accompanied by a substantial reduction in ΔH following collagen incorporation suggests disruption of PVA crystalline domains by the amorphous protein structure, in agreement with previous reports on PVA–collagen systems. This behavior can also be associated with intermolecular hydrogen bonding between the hydroxyl groups of PVA and functional groups present in collagen, which further restricts polymer chain mobility [101]. The incorporation of rGO resulted in a further increase in Tg and partial recovery of ΔH, indicating improved molecular packing and enhanced thermal resistance. This behavior has been attributed in the literature to strong interfacial interactions between rGO nanosheets and polymer chains, which restrict chain mobility and promote thermal rigidity [101, 102]. In the PVA/Col/rGO/Amox/Ibu scaffold, the highest Tg combined with moderate ΔH values suggests a balanced thermal response, where drug incorporation modifies molecular interactions without severely compromising structural order. The two-step degradation profile observed in the TGA/DTG curves is consistent with the typical degradation behavior of PVA-based composites (Fig. 5D, E). The higher moisture loss observed in the PVA/Col scaffold reflects the hydrophilic nature of collagen, while the reduced mass loss in rGO- and drug-containing scaffolds indicates improved thermal stability. The two-step degradation pattern observed in the TGA/DTG curves corresponds to the typical degradation mechanism of PVA-based composites, where the first stage is mainly associated with dehydration of PVA hydroxyl groups and partial degradation of collagen side chains, while the second stage corresponds to the decomposition of the polymer backbone and carbonaceous residues. The decrease in peak decomposition temperature observed with rGO incorporation may be associated with enhanced heat transfer due to the high thermal conductivity of rGO, whereas the broader degradation profile observed after Amox and Ibu incorporation suggests delayed and more gradual thermal decomposition [100]. Additionally, rGO nanosheets may act as a thermal barrier that slows the volatilization of degradation products, further contributing to improved thermal stability of the composite scaffold. This improvement can be attributed to the presence of rGO and other inorganic components, which can act as thermal barriers and slow the volatilization of decomposition products [103, 104]. Overall, the thermal analysis demonstrates that collagen reduces crystallinity and thermal resistance, rGO enhances structural rigidity and thermal stability, and the combined incorporation of Amox and Ibu provides a moderated thermal behavior suitable for maintaining scaffold integrity under physiological and processing conditions.

### Mechanical properties of the 3D biofunctional and conductive neural scaffolds

The mechanical test results indicate that scaffold composition plays a decisive role in determining tensile strength and deformation behavior (Fig. 6). The relatively low strength (3.5 MPa) and elongation (55%) observed for pure PVA are consistent with its hydrophilic nature and limited intermolecular cohesion, which can compromise load-bearing capability under tensile stress [105]. The incorporation of collagen significantly enhanced both tensile strength (6 MPa) and elongation (120%), suggesting improved intermolecular interactions within the polymer matrix. Collagen’s protein backbone likely promotes additional hydrogen bonding and contributes to increased flexibility, leading to a more deformable yet stronger composite structure. Similar improvements in mechanical performance have been reported for PVA–collagen systems in tissue engineering applications [106]. The highest mechanical strength (7 MPa) observed in the PVA/Col/rGO scaffold highlights the reinforcing effect of rGO. The two-dimensional structure of rGO nanosheets can facilitate efficient load transfer, restrict polymer chain mobility, and hinder crack initiation and propagation, thereby improving tensile strength and overall mechanical integrity. These reinforcing mechanisms are widely reported in graphene-based polymer nanocomposites [107]. In the PVA/Col/rGO/Amox/Ibu scaffold, the reduction in maximum tensile stress (5.5 MPa) accompanied by a substantial increase in elongation (150%) at break suggests a shift toward a more ductile and flexible mechanical response [108]. This showed that the addition of amorphous drug molecules enhanced chain mobility and extensibility by regulating polymer–filler interactions [12]. The tensile strength of peripheral nerve tissue has been reported between 1 and 12 MPa, with elongation at break values ranging from 30–150% depending on species and anatomical location [109–111]. The tensile strength of the PVA/Col/rGO/Amox/Ibu scaffold (~5.5 MPa) lies within the reported physiological range of peripheral nerve tissue (approximately 1–12 MPa), positioning it within the lower–mid spectrum of native nerve mechanical properties. Importantly, this value is neither excessively high—which could result in mechanical mismatch and stress shielding—nor too low to compromise structural integrity. Instead, it provides a balanced mechanical profile that ensures sufficient load-bearing capacity while maintaining compliance compatible with soft neural tissues. This mechanical alignment with native peripheral nerve tissue suggests that the scaffold can withstand in vivo mechanical stresses without inducing stiffness-related secondary injury or failure [40, 110, 111]. In particular, the scaffolds were capable of extending to more than 100% of their initial length without rupture, demonstrating their ability to tolerate mechanical deformation and adapt to tensile and compressive stresses at the nerve injury site [109]. Overall, the mechanical analysis demonstrates that collagen improves elasticity, rGO enhances tensile strength, and the combined inclusion of therapeutic agents promotes flexibility, allowing the mechanical properties of the scaffold to be tuned through compositional design [105, 106, 112].

### Degradability analysis of the 3D biofunctional and conductive neural scaffolds

In Fig. 7, the degradation results indicate that scaffold composition strongly influences structural stability under physiological conditions. The rapid degradation of pure PVA is consistent with its high hydrophilicity, high swelling capacity, and lack of crosslinking, which facilitate water penetration, polymer chain mobility, and material dissolution [113]. Although the incorporation of collagen introduces additional intermolecular interactions, the PVA/Col scaffold still exhibited rapid degradation, likely due to the hydrophilic and partially amorphous nature of collagen. The incorporation of rGO contributed to improved structural stability by restricting polymer chain mobility and acting as a physical barrier to water diffusion. Similar stabilizing effects of graphene-based fillers on polymer degradation behavior have been reported in previous studies, where rGO enhances interfacial interactions and limits scaffold disintegration [114]. The most pronounced improvement in degradation resistance was observed in the PVA/Col/rGO/Amox/Ibu scaffold. The reduced weight loss suggests that the integration of amoxicillin and ibuprofen contributes to additional intermolecular interactions and partial pore filling, thereby slowing water ingress and scaffold erosion. This enhanced durability may be beneficial for applications requiring temporary structural support and sustained bioactivity. Overall, these findings demonstrate that scaffold degradation can be effectively tuned through the combined incorporation of rGO and bioactive agents, enabling control over structural persistence in physiological environments [115, 116].

### Controlled drug release from the 3D biofunctional and conductive neural scaffolds

The observed release profiles indicate a biphasic release behavior characterized by an initial burst followed by a slower, sustained release phase. The rapid initial release is commonly attributed to the diffusion of drug molecules weakly adsorbed on or near the scaffold surface, as reported for polymer-based drug delivery systems [112, 117]. The significantly higher cumulative release of Amox compared to Ibu can be explained by differences in their physicochemical properties (Fig. 8A, B). Amoxicillin is a hydrophilic molecule with high aqueous solubility, which facilitates faster diffusion through the hydrated scaffold matrix during the early release phase. In contrast, ibuprofen is a lipophilic drug with limited water solubility, resulting in reduced mobility and stronger retention within the polymer–rGO network. This leads to a delayed and more sustained release profile, consistent with previously reported behavior of lipophilic drugs in composite hydrogel scaffolds [117–119]. Although the release duration of 480 min may appear short compared to long-term implantable systems, early-phase burst release and short-term sustained release are particularly advantageous in neural injury settings, where localized therapeutic concentrations are needed rapidly at the injury site (Fig. 8C) [40]. Following peripheral and spinal cord injuries, the acute phase begins immediately after the primary insult and is characterized by rapid inflammatory cell infiltration, cytokine release, oxidative stress, and secondary tissue degeneration. Macrophages rapidly infiltrate the nerve injury site within hours following trauma and play a central role in orchestrating the inflammatory microenvironment. Modulating macrophage polarization toward the M2 phenotype has been shown to attenuate inflammation, decrease the formation of scar tissue, and accelerate tissue repair. Therefore, early local drug release from the scaffold may contribute to shaping macrophage polarization dynamics during the acute phase of nerve injury, promoting a pro-regenerative immune microenvironment. This early window plays a decisive role in determining the extent of secondary damage and long-term functional recovery [120, 121]. Therefore, timely local delivery of anti-inflammatory and neuroprotective agents during the first hours post-injury is critical for modulating the acute inflammatory cascade and minimizing further neuronal loss. The developed scaffolds designed for nervous system repair have been reported to provide temporally controlled therapeutic release aligned with the pathophysiological stages of neural injury. In this context, the observed release profile of the PVA/Col/rGO/Amox/Ibu scaffold is consistent with the acute therapeutic window following nerve trauma and may contribute to early antibacterial protection and inflammation control at the injury site [40, 120–122]. The dual-drug release behavior observed in this study demonstrates the ability of the PVA/Col/rGO scaffold to simultaneously accommodate and release therapeutic agents with distinct solubility characteristics. Such differential release kinetics may be advantageous for neural tissue engineering applications, where an initial antibacterial effect (Amox) followed by prolonged anti-inflammatory activity (Ibu) is desirable. Overall, these findings confirm that scaffold composition and drug physicochemical properties play a critical role in governing release kinetics from multifunctional 3D-printed systems [83, 84].

### Release kinetics of the 3D biofunctional and conductive neural scaffolds

The kinetic modeling results indicate that drug release from the PVA/Col/rGO/Amox/Ibu scaffold does not follow a simple single-mechanism process (Table 6, Fig. 9). The moderate fit of the Zero-order model suggests that while controlled release may occur during the early stages, the release rate is not constant over time. This deviation is expected in swellable polymeric systems, where matrix hydration and structural rearrangement progressively influence drug diffusion. The poor correlation with the First-order model indicates that release is not governed solely by the concentration gradient of the drug within the scaffold. Instead, physical factors such as polymer swelling, matrix relaxation, and degradation significantly contribute to release behavior. This is particularly relevant in the present system, where hydrophilic PVA and collagen undergo time-dependent swelling in aqueous environments. The strong agreement with the Higuchi model suggests that diffusion plays a dominant role in drug release, especially during the early and intermediate stages. The formation of a hydrated polymeric diffusion pathway due to PVA swelling and rGO-mediated network stabilization supports diffusion-controlled transport. However, the suitability of the Hixson–Crowell model indicates that matrix erosion and dimensional changes also contribute, particularly at prolonged release times when scaffold integrity gradually decreases. The Korsmeyer–Peppas analysis further supports this interpretation. The calculated *n* value falling within the anomalous transport range confirms that drug release is governed by a combination of Fickian diffusion and polymer chain relaxation or swelling. This mixed mechanism is typical of hydrogel-based composite systems incorporating both hydrophilic polymers and nanofillers. The Weibull model provided the best overall fit, reflecting the complex, time-dependent nature of the release process. The high *R*^2^ values and a shape parameter *b* > 1 indicate an initial burst release followed by a slower, sustained diffusion phase, consistent with the experimental release profiles of both drugs. This behavior confirms that the PVA/Col/rGO scaffold enables multiphasic release, which is advantageous for biomedical applications requiring an early therapeutic dose followed by prolonged drug availability.

The kinetic modeling results demonstrate that drug release from the PVA/Col/rGO/Amox/Ibu scaffold is governed by multiple, overlapping mechanisms rather than a single kinetic process. The moderate agreement with the Zero-order model suggests that constant-rate release is not maintained throughout the release period, which is typical for hydrogel-based and swellable polymeric systems. Deviations from Zero-order behavior are commonly attributed to matrix hydration, swelling, and degradation over time. The poor fit of the First-order model, particularly for Amox, indicates that release is not solely concentration-dependent. Instead, structural changes in the polymer matrix, including swelling and degradation of PVA and collagen, play a dominant role. This interpretation is supported by the strong correlation observed with the Higuchi model, confirming diffusion as a primary release mechanism, especially during the early phase. The applicability of the Hixson–Crowell model suggests that matrix erosion contributes to drug release, particularly at prolonged time points where scaffold integrity is reduced. This is consistent with the aqueous degradation behavior of PVA/Col-based systems. The anomalous n values obtained from the Korsmeyer–Peppas model further confirm a non-Fickian transport mechanism, indicating the simultaneous contribution of diffusion and polymer chain relaxation or swelling. Drug-specific differences in release behavior are directly related to physicochemical properties. Amox, being hydrophilic, exhibited release governed by both diffusion and polymer relaxation, as indicated by its best fit with the Korsmeyer–Peppas model. In contrast, Ibu showed predominantly diffusion-controlled release, consistent with its stronger correlation with the Higuchi model and its more hydrophobic nature, which limits rapid mobility within the hydrated matrix. Although the Weibull model showed the highest overall fit for the combined system, its moderate drug-specific correlations suggest that it effectively captures the global release profile but provides limited mechanistic insight. Nevertheless, the Weibull shape parameter (*b* > 1) confirms a biphasic release behavior characterized by an initial burst followed by sustained release. Overall, these findings demonstrate that the developed PVA/Col-based scaffold enables differentiated and time-dependent release of dual therapeutic agents, allowing hydrophilic and hydrophobic drugs to follow distinct yet predictable kinetic pathways. This multi-mechanistic release behavior highlights the potential of the composite system for controlled and tailored drug delivery applications [123–126].

### Antibacterial effect of the 3D biofunctional and conductive neural scaffolds

The antibacterial results clearly demonstrate that the antimicrobial activity of the developed scaffolds is primarily associated with the presence of amoxicillin (Fig. 10). Significant inhibition zones observed for both *E. coli* (gram-negative) and *S. aureus* (gram-positive) confirm the broad-spectrum antibacterial efficacy of amoxicillin when incorporated into the PVA/Col/rGO matrix. Notably, the PVA/Col/rGO/Amox/Ibu scaffold produced a substantially larger inhibition zone against *E. coli* (Fig. 10A) and S. *aureus* (Fig. 10B) compared to the Amox-only scaffold, suggesting a synergistic effect between amoxicillin and ibuprofen. Although ibuprofen is not a classical antibiotic, previous studies have reported its ability to enhance antimicrobial efficacy by altering bacterial membrane permeability and modulating inflammatory microenvironments, which may facilitate antibiotic diffusion and action [75]. The absence of inhibition zones in scaffolds lacking amoxicillin confirms that neither rGO nor ibuprofen alone provides sufficient antibacterial activity at the tested concentrations [127, 128]. This observation highlights the dominant role of amoxicillin in bacterial growth inhibition, while ibuprofen acts as a supportive agent that enhances the overall antimicrobial performance when combined [81]. Overall, the dual-drug-loaded PVA/Col/rGO scaffold demonstrates enhanced and synergistic antibacterial efficacy, making it a promising candidate for neural tissue engineering applications where infection control is critical alongside biofunctionality [82].

### Evaluation of cytocompatibility of the 3D biofunctional and conductive neural scaffolds

The gradual increase in cell viability observed with the addition of collagen, rGO, and bioactive agents highlights the synergistic role of each component in improving scaffold biocompatibility (Fig. 11A). Collagen is known to enhance cell adhesion and spreading due to its native extracellular matrix–like structure, which facilitates integrin-mediated cell attachment [129]. The further improvement observed with rGO incorporation can be attributed to its large surface area, enhanced protein adsorption, and improved electrical and physicochemical interactions at the cell–material interface [130]. Importantly, the inclusion of amoxicillin and ibuprofen did not inhibit cell proliferation. On the contrary, the PVA/Col/rGO/Amox/Ibu scaffold supported the highest and most sustained cell viability over time. This suggests that the antibacterial and anti-inflammatory agents not only preserve cellular compatibility but may also improve long-term cellular adaptation by reducing inflammatory stress and maintaining a favorable microenvironment. The observation that cell viability continued to increase up to day 7, reaching its maximum in the dual-drug-loaded scaffold, indicates that long-term cell–material interactions are stable and supportive of proliferation (Fig. 11B). The comparable or superior viability relative to TCP further confirms the cytocompatibility of the developed scaffolds and their suitability for tissue engineering applications. These findings are consistent with previous reports demonstrating high biocompatibility of PVA-based composite scaffolds. For example, a similar study using a PVA/GO/Col scaffold reported 100% viability and non-toxic behavior toward human keratinocyte (HaCaT) cells [131]. Collectively, the results confirm that the developed 3D biofunctional and conductive neural scaffolds are non-cytotoxic and capable of promoting sustained cell proliferation, with the PVA/Col/rGO/Amox/Ibu formulation showing particular promise for advanced biomedical and neural tissue engineering applications [129, 130].

## Conclusion

In this study, a 3D biofunctional and conductive neural scaffold composed of PVA, collagen, reduced graphene oxide, amoxicillin, and ibuprofen was successfully developed for neural tissue engineering applications. The developed 3D scaffold exhibited excellent printability, ensuring reproducibility and structural integrity during the extrusion-based printing process. The incorporation of rGO significantly enhanced the electrical conductivity of the scaffold, forming an interconnected conductive network favorable for neural regeneration. Morphological analysis confirmed the porous architecture suitable for cell infiltration and nutrient exchange. Mechanical testing revealed that the scaffold possessed sufficient strength and elasticity to support neural tissue regeneration. Additionally, degradation tests demonstrated an appropriate degradation rate compatible with extracellular matrix remodeling during tissue repair. Moreover, the dual-drug loading enabled a controlled release profile, with amoxicillin exhibiting rapid release due to its hydrophilic nature, and ibuprofen showing a more sustained release attributed to its lipophilicity. Kinetic modeling indicated that the drug release profile best fit the Weibull model. These findings collectively demonstrate the potential of this multifunctional scaffold to provide both structural support and bioelectrical cues essential for neural tissue repair. The scaffold also exhibited significant antibacterial activity, especially attributed to the synergistic effect of Amox and rGO against common pathogens, indicating its potential to prevent post-implantation infections. Cytotoxicity analysis using the MTT assay confirmed that the scaffold is biocompatible and supports cell viability, making it a promising candidate for further biological evaluation. Overall, the biofunctional and conductive properties, combined with printability, mechanical integrity, biodegradability, antibacterial performance, and cytocompatibility, highlight the great potential of this scaffold system for future applications in neural tissue engineering. Future in vitro and in vivo investigations are warranted to comprehensively assess the scaffold’s long-term functionality, biocompatibility, and therapeutic efficacy in neural tissue regeneration.

## Data Availability

The authors confirm that the data supporting the findings of this study are available within the article. Raw data that support the findings of this study are available from the corresponding author upon reasonable request.

## Code availability

The authors confirm that the data supporting the findings of this study are available within the article. Raw data that support the findings of this study are available from the corresponding author upon reasonable request.

## References

[1] Park J, Jeon J, Kim B, Lee MS, Park S, Lim J, et al. Electrically conductive hydrogel nerve guidance conduits for peripheral nerve regeneration. Adv Funct Mater. 2020;30:1–14. 10.1002/adfm.202003759.

[2] Sarhane KA, Qiu C, Harris TGW, Hanwright PJ, Mao HQ, Tuffaha SH. Translational bioengineering strategies for peripheral nerve regeneration: opportunities, challenges, and novel concepts. Neural Regen Res. 2023;18:1229–34. 10.4103/1673-5374.358616. 36453398 10.4103/1673-5374.358616 PMC9838159

[3] Chen X, Ranjan VD, Liu S, Liang YN, Lim JSK, Chen H, et al. In situ formation of 3D conductive and cell-laden graphene hydrogel for electrically regulating cellular behavior. Macromol Biosci. 2021;21:1–11. 10.1002/mabi.202000374. 10.1002/mabi.202000374 33620138

[4] Mansouri N, Al-Sarawi S, Losic D, Mazumdar J, Clark J, Gronthos S, et al. Biodegradable and biocompatible graphene-based scaffolds for functional neural tissue engineering: a strategy approach using dental pulp stem cells and biomaterials. Biotechnol Bioeng. 2021;118:4217–30. 10.1002/bit.27891. 34264518 10.1002/bit.27891

[5] Talebi A, Labbaf S, Rahmati S. Biofabrication of a flexible and conductive 3D polymeric scaffold for neural tissue engineering applications; physical, chemical, mechanical, and biological evaluations. Polym Adv Technol. 2023;34:134–44. 10.1002/pat.5872.

[6] Katoli Z, Navaei-Nigjeh M, Mirzababaei S, Sabahi H, Baeeri M, Akrami M, et al. Incorporation of montmorillonite into microfluidics-generated chitosan microfibers enhances neuron-like PC12 cells for application in neural tissue engineering. Carbohydr Polym. 2024;342:122272. 10.1016/j.carbpol.2024.122272. 39048184 10.1016/j.carbpol.2024.122272

[7] Lotfi L, Khakbiz M, Moosazadeh Moghaddam M, Bonakdar S. A biomaterials approach to Schwann cell development in neural tissue engineering. J Biomed Mater Res Part A. 2019;107:2425–46. 10.1002/jbm.a.36749. 10.1002/jbm.a.36749 31254439

[8] Kohestani AA, Xu Z, Baştan FE, Boccaccini AR, Pishbin F. Electrically conductive coatings in tissue engineering. Acta Biomater. 2024;186:30–62. 10.1016/j.actbio.2024.08.007. 39128796 10.1016/j.actbio.2024.08.007

[9] Raikar AS, Kalaskar DM, Bhilegaonkar S, Somnache SN, Bodaghi M. Revolutionizing drug delivery by bioinspired 4D transdermal microneedles: advances and future horizons. Eur Polym J. 2024;210:112952. 10.1016/j.eurpolymj.2024.112952.

[10] Li Q, Ma L, Gao C. Biomaterials for in situ tissue regeneration: Development and perspectives. J Mater Chem B. 2015;3:8921–38. 10.1039/c5tb01863c. 32263026 10.1039/c5tb01863c

[11] Kashte SB. Synthesis and evaluation of a novel combination scaffold and its application in bone tissue engineering. Kolhapur: D. Y. Patil Education Society (Deemed to be University); 1956.

[12] Nagarajan A, Rizwana N, Abraham M, Bhat M, Vetekar A, Thakur G, et al. Polycaprolactone/graphene oxide/acellular matrix nano fibrous scaffolds with antioxidant and promyelinating features for the treatment of peripheral demyelinating diseases. J Mater Sci Mater Med. 2023;34:49. 10.1007/s10856-023-06750-2. 37796399 10.1007/s10856-023-06750-2 PMC10556163

[13] Chakraborty P, Oved H, Bychenko D, Yao Y, Tang Y, Zilberzwige-Tal S, et al. Nanoengineered peptide-based antimicrobial conductive supramolecular biomaterial for cardiac tissue engineering. Adv Mater. 2021;33:1–10. 10.1002/adma.202008715. 10.1002/adma.202008715 34033154

[14] Kharaziha M, Shin SR, Nikkhah M, Topkaya SN, Masoumi N, Annabi N, et al. Tough and flexible CNT-polymeric hybrid scaffolds for engineering cardiac constructs. Biomaterials. 2014;35:7346–54. 10.1016/j.biomaterials.2014.05.014. 24927679 10.1016/j.biomaterials.2014.05.014 PMC4114042

[15] Gungor-Ozkerim PS, Inci I, Zhang YS, Khademhosseini A, Dokmeci MR. Bioinks for 3D bioprinting: an overview. Biomater Sci. 2018;6:915–46. 10.1039/c7bm00765e. 29492503 10.1039/c7bm00765e PMC6439477

[16] Cunha C, Panseri S, Antonini S. Emerging nanotechnology approaches in tissue engineering for peripheral nerve regeneration. Nanomed Nanotechnol Biol Med. 2011;7:50–59. 10.1016/j.nano.2010.07.004. 10.1016/j.nano.2010.07.004 20692373

[17] Rizwana N, Maslekar N, Chatterjee K, Yao Y, Agarwal V, Nune M. Dual crosslinked antioxidant mixture of poly (vinyl alcohol) and cerium oxide nanoparticles as a bioink for 3D bioprinting. 2024. 10.1021/acsanm.3c02962. 10.1021/acsanm.3c02962 PMC11348314 39206348

[18] Kose A, Altan E, Abdulazez IF, Bingol AB, Yılmaz H, Erarslan A, et al. Design and production of biofunctional PVA/PLA double-layered fiber wound dressing by electrospinning method. J Appl Polym Sci. 2025. 10.1002/app.57067.

[19] Gao C, Song S, Lv Y, Huang J, Zhang Z. Recent development of conductive hydrogels for tissue engineering: review and perspective. Macromol Biosci. 2022;22:1–24. 10.1002/mabi.202200051. 10.1002/mabi.202200051 35472125

[20] Abzan N, Kharaziha M, Labbaf S. Development of three-dimensional piezoelectric polyvinylidene fluoride-graphene oxide scaffold by non-solvent induced phase separation method for nerve tissue engineering. Mater Des. 2019;167:107636. 10.1016/j.matdes.2019.107636.

[21] Abedi A, Bakhshandeh B, Babaie A, Mohammadnejad J, Vahdat S, Mombeiny R, et al. Concurrent application of conductive biopolymeric chitosan/ polyvinyl alcohol/MWCNTs nanofibers, intracellular signaling manipulating molecules and electrical stimulation for more effective cardiac tissue engineering. Mater Chem Phys. 2021;258:123842. 10.1016/j.matchemphys.2020.123842.

[22] Shrestha S, Shrestha BK, Lee J, Joong OK, Kim BS, Park CH, et al. A conducting neural interface of polyurethane/silk-functionalized multiwall carbon nanotubes with enhanced mechanical strength for neuroregeneration. Mater Sci Eng C. 2019;102:511–23. 10.1016/j.msec.2019.04.053. 10.1016/j.msec.2019.04.053 31147022

[23] Magaz A, Li X, Gough JE, Blaker JJ. Graphene oxide and electroactive reduced graphene oxide-based composite fibrous scaffolds for engineering excitable nerve tissue. Mater Sci Eng C. 2021. 10.1016/j.msec.2020.111632. 10.1016/j.msec.2020.111632 33321671

[24] Arun Karthick S, Ragavi TK, Naresh K, Rama Sreekanth PS. A study on collagen-PVA and chitosan-PVA nanofibrous matrix for wound dressing application. Mater Today Proc. 2022;56:1347–50. 10.1016/j.matpr.2021.11.421.

[25] Almuqoddas E, Hambyah I, Rizqiyanti R, Subagio A, Umiati NAK. Characterisation of collagen from the skin of catfish (Pangasius sp) for innovative PVA-collagen nanofiber. E3S Web Conf. 2019;125:9. 10.1051/e3sconf/201912504002.

[26] Keirouz A, Mustafa YL, Turner JG, Lay E, Jungwirth U, Marken F, et al. Conductive polymer-coated 3D printed microneedles: biocompatible platforms for minimally invasive biosensing interfaces. Small. 2023;19:e2206301. 10.1002/smll.202206301. 36596657 10.1002/smll.202206301

[27] Dong R, Ma PX, Guo B. Conductive biomaterials for muscle tissue engineering. Biomaterials. 2020. 10.1016/j.biomaterials.2019.119584. 10.1016/j.biomaterials.2019.119584 31704468

[28] Xu Y, Patino Gaillez M, Rothe R, Hauser S, Voigt D, Pietzsch J, et al. Conductive hydrogels with dynamic reversible networks for biomedical applications. Adv Healthc Mater. 2021;10:e2100012. 10.1002/adhm.202100012. 33930246 10.1002/adhm.202100012 PMC11468162

[29] Tomczykowa M, Plonska-Brzezinska ME. Conducting polymers, hydrogels and their composites: preparation, properties and bioapplications. Polymers. 2019;11:1–36. 10.3390/polym11020350. 10.3390/polym11020350 PMC6419165 30960334

[30] Abdulazez IF, Oktay B, Yilmaz H, Betul A. Design and development of rGO and polymer- ­ based patches for cardiac tissue engineering. J Appl Polym Sci. 2025. 10.1002/app.57767.

[31] Athukorala SS, Tran TS, Balu R, Truong VK, Chapman J, Dutta NK, et al. 3D printable electrically conductive hydrogel scaffolds for biomedical applications: a review. Polymers. 2021;13:1–21. 10.3390/polym13030474. 10.3390/polym13030474 PMC7867335 33540900

[32] Zarrintaj P, Zangene E, Manouchehri S, Amirabad LM, Baheiraei N, Hadjighasem MR, et al. Conductive biomaterials as nerve conduits: recent advances and future challenges. Appl Mater Today. 2020;20:100784. 10.1016/j.apmt.2020.100784.

[33] Mindivan F. A comparison study on mechanical properties of PVC composites filled by graphene oxide (GO) and reduced graphene oxide (RGO). Pamukkale Univ J Eng Sci. 2019;25:43–48. 10.5505/pajes.2018.39114.

[34] Sabzehmeidani MM, Mahnaee S, Ghaedi M, Heidari H, Roy VAL. Carbon based materials: a review of adsorbents for inorganic and organic compounds,. Mater Adv. 2021;2:598–627. 10.1039/d0ma00087f.

[35] Rogers ZJ, Zeevi MP, Koppes R, Bencherif SA. Electroconductive Hydrogels for tissue engineering: current status and future perspectives. Bioelectricity. 2020;2:279–92. 10.1089/bioe.2020.0025. 34476358 10.1089/bioe.2020.0025 PMC8370338

[36] Suzuki H, Imajo Y, Funaba M, Ikeda H, Nishida N. Current concepts of biomaterial scaffolds and regenerative therapy for spinal cord Injury. Int J Mol Sci. 2023;24:2528. 36768846 10.3390/ijms24032528 PMC9917245

[37] Zhai Y, Guan X, Lu C, Sun R, Qian Y, Li Y, et al. Injectable chitosan-based hydrogel via in situ gelation modulates the inflammatory microenvironment and facilitates minimally invasive repair of peripheral nerve injury. Mater Today Bio. 2026;37:102814. 10.1016/j.mtbio.2026.102814. 41624516 10.1016/j.mtbio.2026.102814 PMC12859466

[38] Cano-vicent A, El-tanani M, Tambuwala MM, Mishra YK. Scaffolds in the microbial resistant era: fabrication, materials, properties and tissue engineering applications. Mater Today Bio. 2022;16:100412. 10.1016/j.mtbio.2022.100412. 10.1016/j.mtbio.2022.100412 PMC9463390 36097597

[39] Luca IM, Earar K, Chiuariu TM, Dobri VS, Trifautanu P, Ilie M, et al. Non-steroidal anti-inflammatory agents in temporomandibular joint disorders: current evidence and therapeutic perspectives. Rom J Med Dent Educ. 2025;14:103–9.

[40] Kellaway SC, Ullrich MM, Dziemidowicz K. Electrospun drug-loaded scaffolds for nervous system repair. Wiley Interdiscip Rev Nanomed Nanobiotechnol. 2024. 10.1002/wnan.1965. 10.1002/wnan.1965 38740385

[41] Dziemidowicz K, Kellaway SC, Guillemot-Legris O, Matar O, Trindade RP, Roberton VH, et al. Development of ibuprofen-loaded electrospun materials suitable for surgical implantation in peripheral nerve injury. Biomater Adv. 2023;154:213623. 10.1016/j.bioadv.2023.213623. 37837905 10.1016/j.bioadv.2023.213623

[42] Liu X, Miller AL 2nd, Park S, George MN, Waletzki BE, Xu H, et al. Two-dimensional black phosphorus and graphene oxide nanosheets synergistically enhance cell proliferation and osteogenesis on 3D printed scaffolds. ACS Appl Mater Interfaces. 2019;11:23558–72. 10.1021/acsami.9b04121. 31199116 10.1021/acsami.9b04121 PMC8942345

[43] Wang S, Guan S, Sun C, Liu H, Liu T, Ma X. Electrical stimulation enhances the neuronal differentiation of neural stem cells in three-dimensional conductive scaffolds through the voltage-gated calcium ion channel. Brain Res. 2023;1798:148163. 10.1016/j.brainres.2022.148163. 36379314 10.1016/j.brainres.2022.148163

[44] Rinoldi C, Lanzi M, Fiorelli R, Nakielski P, Zembrzycki K, Kowalewski T, et al. Three-dimensional printable conductive semi-interpenetrating polymer network hydrogel for neural tissue applications. Biomacromolecules. 2021;22:3084–98. 10.1021/acs.biomac.1c00524. 34151565 10.1021/acs.biomac.1c00524 PMC8462755

[45] Wang J, Wang H, Mo X, Wang H. Reduced graphene oxide-encapsulated microfiber patterns enable controllable formation of neuronal-like networks. Adv. Mater. 2020;32:2004555. 10.1002/adma.202004555. 10.1002/adma.202004555 PMC10865229 32875631

[46] Zhao Y, Liu Y, Lu C, Sun D, Kang S, Wang X, et al. Reduced graphene oxide fibers combined with electrical stimulation promote peripheral nerve regeneration. Int J Nanomed. 2024;19:2341–57. 10.2147/IJN.S449160 PMC10926921 38469057

[47] Convertino D, Trincavelli ML, Giacomelli C, Marchetti L, Coletti C. Graphene-based nanomaterials for peripheral nerve regeneration. Front Bioeng Biotechnol. 2023. 10.3389/fbioe.2023.1306184. 10.3389/fbioe.2023.1306184 PMC10757979 38164403

[48] Bian T, Jiang Y, Cao J, Wu W, Zhang L, Yang Y. Fabrication of piezoelectric/conductive composite nerve conduits for peripheral nerve regeneration. Colloids Surf B Biointerfaces. 2025;250:114544. 10.1016/j.colsurfb.2025.114544. 39983450 10.1016/j.colsurfb.2025.114544

[49] Verbanic S, Lee J, Deacon JM, Chen IA. Microbial predictors of healing and short-term effect of debridement on the microbiome of chronic wounds. npj Biofilms Microbiomes. 2020. 10.1038/s41522-020-0130-5. 10.1038/s41522-020-0130-5 PMC7195478 32358500

[50] Oktay A, Yilmazer H, Przekora A, Yilmazer Y, Wojcik M, Dikici B, et al. Corrosion response and biocompatibility of graphene oxide (GO)–serotonin (Ser) coatings on Ti6Al7Nb and Ti29Nb13Ta4.6Zr (TNTZ) alloys fabricated by electrophoretic deposition (EPD). Mater Today Commun. 2023;34:105236. 10.1016/j.mtcomm.2022.105236.

[51] Agarwal V, Zetterlund PB. Strategies for reduction of graphene oxide – a comprehensive review. Chem Eng J. 2021;405:127018.

[52] Mauricio J, Mancipe A, Dias ML, Mara R, Silva D. Type I collagen – poly (vinyl alcohol) electrospun nanofibers: FTIR study of the collagen helical structure preservation. Polym Technol Mater. 2022;61:846–60. 10.1080/25740881.2022.2029887.

[53] Alam R, Alimuzzaman S, Shahid A. Collagen / Nigella sativa / chitosan inscribed electrospun hybrid bio-nanocomposites for skin tissue engineering. J Biomater Sci Polym Ed. 2023;34:1517–38. 10.1080/09205063.2023.2170139. 36779683 10.1080/09205063.2023.2170139

[54] Manzari-Tavakoli A, Tarasi R, Sedghi R, Moghimi A, Niknejad H. Fabrication of nanochitosan incorporated polypyrrole/alginate conducting scaffold for neural tissue engineering. Sci Rep. 2020;10:1–10. 10.1038/s41598-020-78650-2. 33328579 10.1038/s41598-020-78650-2 PMC7744540

[55] Jeong SI, Jun ID, Choi MJ, Nho YC, Lee YM, Shin H. Development of electroactive and elastic nanofibers that contain polyaniline and poly(L-lactide-co-ε-caprolactone) for the control of cell adhesion. Macromol Biosci. 2008;8:627–37. 10.1002/mabi.200800005. 18401867 10.1002/mabi.200800005

[56] Özge B, Ergür Ö, Ciftci F, Ahlatcıo E. Production and characterization of conductive scaffolds for conducting tissue engineering. ChemistrySelect. 2025. 10.1002/slct.202500790.

[57] Naghieh S, Sarker MD, Sharma NK, Barhoumi Z, Chen X. Printability of 3D printed hydrogel scaffolds: influence of hydrogel composition and printing parameters. Appl Sci. 2020. 10.3390/app10010292.

[58] Testore D, Zoso A, Kortaberria G, Sangermano M, Chiono V. Electroconductive photo-curable PEGDA-gelatin/PEDOT:PSS hydrogels for prospective cardiac tissue engineering application. Front Bioeng Biotechnol. 2022;10:1–14. 10.3389/fbioe.2022.897575. 10.3389/fbioe.2022.897575 PMC9263513 35814009

[59] Postolović K, Ljujić B, Kovačević MM, Đorđević S, Nikolić S, Živanović S, et al. Optimization, characterization, and evaluation of carrageenan/alginate/poloxamer/curcumin hydrogel film as a functional wound dressing material. Mater Today Commun. 2022;31:103528. 10.1016/j.mtcomm.2022.103528.

[60] Ozder MN, Ciftci F, Rencuzogullari O, Arisan ED, Ustündag CB. In situ synthesis and cell line studies of nano-hydroxyapatite/graphene oxide composite materials for bone support applications. Ceram Int. 2023;49:14791–803. 10.1016/j.ceramint.2023.01.075.

[61] Paper C, Palm C, Mill O, Bhd S, Liu WW. Comparison on graphite, graphene oxide and reduced graphene oxide: synthesis and characterization. AIP Conf Proc. 2017. 10.1063/1.5005764.

[62] Habte AT, Ayele DW. Synthesis and characterization of reduced graphene oxide (rGO) started from graphene oxide (GO) using the tour method with different parameters. Adv Mater Sci Eng. 2019;2019:5058163.

[63] Fan B, Guo H, Shi J, Shi C, Jia Y, Wang H. Facile one-pot preparation of silver / reduced graphene oxide nanocomposite for cancer photodynamic and photothermal therapy. J Nanosci Nanotechnol. 2016. 10.1166/jnn.2016.11327.

[64] Fentaw T, Worku D. Controlled synthesis, characterization and reduction of graphene oxide: a convenient method for large scale production. Egypt J Basic Appl Sci. 2021. 10.1016/j.ejbas.2016.11.002.

[65] Altınay E, Köse FZ, Ateş SC, Kızılbey K. Ibuprofen-loaded silver nanoparticle-doped PVA gels: green synthesis, in vitro cytotoxicity, and antibacterial analyses. Gels. 2024;10:143. 10.3390/gels10020143 PMC10887808 38391473

[66] García-Hernández AB, Morales-Sánchez E, Berdeja-Martínez BM, Escamilla-García M, Salgado-Cruz MP, Rentería-Ortega M, et al. PVA-based electrospun biomembranes with hydrolyzed collagen and ethanolic extract of hypericum perforatum for potential use as wound dressing: fabrication and characterization. Polymers. 2022;14:1981. 10.3390/polym14101981 PMC9147280 35631864

[67] Çerçi A, Sena E, Esra D, Ça K, Güzel B, Osman B. Preparation and characterization of amoxicillin-loaded polyvinyl alcohol/sodium alginate nanofibrous mat: drug release properties, antibacterial activity, and cytotoxicity. Arab J Sci Eng. 2025;50:77–91. 10.1007/s13369-024-09075-6.

[68] Salavagione HJ, Martínez G, Gómez MA. Synthesis of poly (vinyl alcohol)/reduced graphite oxide nanocomposites with improved thermal and electrical properties. J Mater Chem. 2009;19:5027–32. 10.1039/b904232f.

[69] Ciobanu SC, Predoi D, Iconaru SL, Rokosz K, Raaen S, Bleotu C, et al. Development of chrome-doped hydroxyapatite in a PVA matrix enriched with amoxicillin for biomedical applications. Antibiotics. 2025;14:455. 40426522 10.3390/antibiotics14050455 PMC12108204

[70] Bingol AB, Karakas CY, Akkurt Yildirim M, Insel MA, Zaman AC, Oktay B, et al. Development of a potential multilayered biofunctional dressing for localized postoperative cancer treatment: a hybrid approach using 3D printing and electrospinning. Macromol Mater Eng. 2025. 10.1002/mame.202500218.

[71] Tran DT, Nguyen VN. RGO/persulfate metal-free catalytic system for the degradation of tetracycline: effect of reaction parameters. Mater Res Express. 2020;7:75501. 10.1088/2053-1591/ab9e47.

[72] Zhu X, Xu X, Liu F, Jin J, Liu L, Zhi Y, et al. Green synthesis of graphene nanosheets and their in vitro cytotoxicity against human prostate cancer (DU 145) cell lines. Nanomater Nanotechnol. 2017;7:1–7. 10.1177/1847980417702794.

[73] Gong Y, Li D, Fu Q, Pan C. Influence of graphene microstructures on electrochemical performance for supercapacitors. Prog Nat Sci Mater Int. 2015;25:379–85. 10.1016/j.pnsc.2015.10.004.

[74] Sengupta I, Chakraborty S, Talukdar M, Pal SK, Chakraborty S. Thermal reduction of graphene oxide: how temperature influences purity. J Mater Res. 2018;33:4113–22. 10.1557/jmr.2018.338.

[75] Glover K, Mathew E, Pitzanti G, Magee E, Lamprou DA. 3D bioprinted scaffolds for diabetic wound-healing applications. Drug Deliv Transl Res. 2023;13:2096–109. 10.1007/s13346-022-01115-8. 35018558 10.1007/s13346-022-01115-8 PMC10315349

[76] Karavas E, Koutris E, Papadopoulos AG, Sigalas MP, Nanaki S, Papageorgiou GZ, et al. Application of density functional theory in combination with FTIR and DSC to characterise polymer drug interactions for the preparation of sustained release formulations between fluvastatin and carrageenans. Int J Pharm. 2014;466:211–22. 10.1016/j.ijpharm.2014.02.049. 24613179 10.1016/j.ijpharm.2014.02.049

[77] Thamilselvan G, David H, Sajeevan A, Rajaramon S, Solomon AP, Durai RD, et al. Polymer based dual drug delivery system for targeted treatment of fluoroquinolone resistant Staphylococcus aureus mediated infections. Sci Rep. 2023;13:1–19. 10.1038/s41598-023-38473-3. 37452106 10.1038/s41598-023-38473-3 PMC10349073

[78] He Q, Fang YQ, Han Y, Qin WN, Nie JK, Hou D, et al. Novel synthesis of PVA/NaCl hydrogel for reversible thermochromism. Opt Mater. 2022;132:112754. 10.1016/j.optmat.2022.112754.

[79] Sun TW, Zhu YJ, Chen F. Hydroxyapatite nanowire/collagen elastic porous nanocomposite and its enhanced performance in bone defect repair. RSC Adv. 2018;8:26218–29. 10.1039/c8ra03972k. 35541968 10.1039/c8ra03972k PMC9082774

[80] Soomro SA, Gul IH, Naseer H, Marwat S, Mujahid M. Improved performance of CuFe2O4/rGO nanohybrid as an anode material for lithium-ion batteries prepared via facile one-step method. Curr Nanosci. 2018;15:420–9. 10.2174/1573413714666181115122016.

[81] Kang Y, Liu J, Wu J, Yin Q, Liang H, Chen A, et al. Graphene oxide and reduced graphene oxide induced neural pheochromocytoma-derived PC12 cell lines apoptosis and cell cycle alterations via the ERK signaling pathways. Int J Nanomedicine. 2017;12:5501–10. 10.2147/IJN.S141032 PMC5546784 28814866

[82] Usala E, Gonzalez Z, Campillo N, Baena J, Rincón E, Ferrari B, et al. Development of 3D printable conductive cellulose-based hydrogel with incorporation of rGO for neural tissue engineering. J Colloid Interface Sci. 2026;703:139285. 10.1016/j.jcis.2025.139285. 10.1016/j.jcis.2025.139285 41129918

[83] Hu W, Liu S, Wang Z, Feng X, Gao M, Song F. In situ reduced graphene oxide and polyvinyl alcohol nanocomposites with enhanced multiple properties. Front Chem. 2022;10:1–10. 10.3389/fchem.2022.856556. 10.3389/fchem.2022.856556 PMC8980314 35392418

[84] Wang X, Su M, Liu C, Shen C, Liu X. Poly (vinyl alcohol)/graphene nanocomposite hydrogel scaffolds for control of cell adhesion. J Renew Mater. 2020;8:89–99. 10.32604/jrm.2020.08493.

[85] Li YM, Ji Y, Meng YX, Kim YJ, Lee H, Kurian AG, et al. Neural tissue-like, not supraphysiological, electrical conductivity stimulates neuronal lineage specification through calcium signaling and epigenetic modification. Adv Sci. 2024;11:e2400586. 10.1002/advs.202400586. 10.1002/advs.202400586 PMC11425260 38984490

[86] Zarrintaj P, Bakhshandeh B, Rezaeian I, Heshmatian B, Ganjali MR. A novel electroactive agarose-aniline pentamer platform as a potential candidate for neural tissue engineering. Sci. Rep. 2017;7:17187. 10.1038/s41598-017-17486-9. 10.1038/s41598-017-17486-9 PMC5719440 29215076

[87] Bagher Z, Atoufi Z, Alizadeh R, Farhadi M, Zarrintaj P, Moroni L, et al. Conductive hydrogel based on chitosan-aniline pentamer/gelatin/agarose significantly promoted motor neuron-like cells differentiation of human olfactory ecto-mesenchymal stem cells. Mater Sci Eng C. 2019;101:243–53. 10.1016/j.msec.2019.03.068. 10.1016/j.msec.2019.03.068 31029317

[88] Wang G, Yang J, Park J, Gou X, Wang B, Liu H, et al. Facile synthesis and characterization of graphene nanosheets. J Phys Chem C. 2008;112:8192–5. 10.1021/jp710931h.

[89] Wang S, Guan S, Li W, Ge D, Xu J, Sun C, et al. 3D culture of neural stem cells within conductive PEDOT layer-assembled chitosan/gelatin scaffolds for neural tissue engineering. Mater Sci Eng C. 2018;93:890–901. 10.1016/j.msec.2018.08.054. 10.1016/j.msec.2018.08.054 30274126

[90] Babai A, Bakhshandeh B, Abedi A, Mohammadnejad J, Shabani I, A Ardeshirylajimi, et al. Synergistic effects of conductive PVA / PEDOT electrospun scaffolds and electrical stimulation for more effective neural tissue engineering, Eur Polym J. 2020. 10.1016/j.eurpolymj.2020.110051.

[91] Ghosh S, Shrivastava A, Jha P, Roy P, Lahiri D. Analysis of neural cell behaviour on anisotropic electrically conductive polymeric biodegradable scaffolds reinforced with carbon nanotubes. Med Devices Sens. 2021. 10.1002/mds3.10152.

[92] Burnstine-Townley A, Eshel Eshel, Amdursky N. Conductive scaffolds for cardiac and neuronal tissue engineering: governing factors and mechanisms. Adv Funct Mater. 2019. 10.1002/adfm.201901369.

[93] Zhao Y, Niu C, Shi J, Wang Y, Yang Y, Wang H. Novel conductive polypyrrole/silk fibroin scaffold for neural tissue repair. Neural Regen Res. 2018. 10.4103/1673-5374.235303. 10.4103/1673-5374.235303 PMC6108196 30106059

[94] Thunberg J, Kalogeropoulos T, Kuzmenko V, Hägg D, Johannesson S, Westman G, et al. In situ synthesis of conductive polypyrrole on electrospun cellulose nanofibers: scaffold for neural tissue engineering. Cellulose. 2015. 10.1007/s10570-015-0591-5.

[95] El-Borady OM. Wastewater treatment using innovative green-synthesized rGO, TiO_2_NPs, and rGO/TiO_2_ nanocomposite: structural, morphological, spectroscopic, thermal, and photocatalytic studies. Water Air Soil Pollut. 2025;236:1–18. 10.1007/s11270-024-07692-3.

[96] Shabil Sha M, Anwar H, Musthafa FN, Al-Lohedan H, Alfarwati S, Rajabathar JR, et al. Photocatalytic degradation of organic dyes using reduced graphene oxide (rGO). Sci Rep. 2024;14:1–14. 10.1038/s41598-024-53626-8. 38351100 10.1038/s41598-024-53626-8 PMC10864344

[97] Stobinski L, Lesiak B, Malolepszy A, Mazurkiewicz M, Mierzwa B, Zemek J, et al. Graphene oxide and reduced graphene oxide studied by the XRD, TEM and electron spectroscopy methods. J Electron Spectros Relat Phenom. 2014;195:145–54. 10.1016/j.elspec.2014.07.003.

[98] Mansouri N, Al-Sarawi SF, Mazumdar J, Losic D. Advancing fabrication and properties of three-dimensional graphene-alginate scaffolds for application in neural tissue engineering. RSC Adv. 2019;9:36838–48. 10.1039/c9ra07481c. 35539075 10.1039/c9ra07481c PMC9075535

[99] Kanayama I, Miyaji H, Takita H, Nishida E, Tsuji M, Fugetsu B, et al. Comparative study of bioactivity of collagen scaffolds coated with graphene oxide and reduced graphene oxide. Int J Nanomed. 2014;9:3363–73. 10.2147/IJN.S62342. 10.2147/IJN.S62342 PMC4103921 25050063

[100] Bahrami S, Baheiraei N, Shahrezaee M. Biomimetic reduced graphene oxide coated collagen scaffold for in situ bone regeneration. Sci Rep. 2021;11:1–10. 10.1038/s41598-021-96271-1. 34408206 10.1038/s41598-021-96271-1 PMC8373942

[101] Singh R, Roopmani R, Hasan U, Dogra P, Giri J. Airbrushed nanofibers with bioactive core and antibacterial shell for wound healing application. Eur. J. Pharm. Biopharm. 2024, 10.1016/j.ejpb.2023.12.009. 10.1016/j.ejpb.2023.12.009 38159872

[102] Choudhury AJ, Gogoi D, Chutia J, Kandimalla R, Kalita S, Kotoky J, et al. Controlled antibiotic-releasing Antheraea assama silk fibroin suture for infection prevention and fast wound healing. Surg (U S). 2016;159:539–47. 10.1016/j.surg.2015.07.022. 10.1016/j.surg.2015.07.022 26328475

[103] Zhang B, He J, Shi M, Liang Y, Guo B. Injectable self-healing supramolecular hydrogels with conductivity and photo-thermal antibacterial activity to enhance complete skin regeneration. Chem Eng J. 2020. 10.1016/j.cej.2020.125994.

[104] González L, Ruiz I, Raposo M, Aguayo C, Toledo JR, Perez-Puyana VM, et al. Collagen/rGO/tannin hydrogels with a programmable biointerface for tunable electrical conductivity and antioxidant capacity in tissue regeneration. Colloids Surf B Biointerfaces. 2026. 10.1016/j.colsurfb.2025.115176. 10.1016/j.colsurfb.2025.115176 41033249

[105] Ju MS, Lin CCK, Chang CT. Researches on biomechanical properties and models of peripheral nerves - a review. J Biomech Sci Eng. 2017;12:1–12. 10.1299/jbse.16-00678.

[106] Grewal R, Xu J, Sotereanos DG, Woo SLY. Biomechanical properties of peripheral nerves. Hand Clin. 1996;12:195–204. 10.1016/s0749-0712(21)00304-8. 8724573

[107] Lee SJ, Yoon SJ, Jeon I. Graphene/polymer nanocomposites: preparation, mechanical properties, and applications. Polymers. 2022;14:4733. 10.3390/polym14214733 PMC9655120 36365726

[108] Hamed Mashhadzadeh A, Hamed Mashhadzadeh A, Golman B, Spitas C, Faroughi SA, Kostas KV. Recent advancements in mechanical properties of graphene-enhanced polymer nanocomposites: progress, challenges, and pathways forward. J Mol Graph Model. 2025;135:108908. 10.1016/j.jmgm.2024.108908. 39579712 10.1016/j.jmgm.2024.108908

[109] Valentino C, Vigani B, Zucca G, Ruggeri M, Marrubini G, Boselli C, et al. Design of novel mechanically resistant and biodegradable multichannel platforms for the treatment of peripheral nerve injuries. Biomacromolecules. 2023. 10.1021/acs.biomac.2c01498. 10.1021/acs.biomac.2c01498 PMC10091422 36922716

[110] Yu L, Bennett CJ, Lin C, Yan S, Yang J. Scaffold design considerations for peripheral nerve regeneration. J Neural Eng. 2025. 10.1088/1741-2552/ad628d.Scaffold. 10.1088/1741-2552/ad628d PMC11883895 38996412

[111] Li G, Kong Y, Zhao Y, Zhao Y, Zhang L, Yang Y. Fabrication and characterization of polyacrylamide/silk fibroin hydrogels for peripheral nerve regeneration. J Biomater Sci Polym Ed. 2015;5063:1–18. 10.1080/09205063.2015.1066109. 10.1080/09205063.2015.1066109 26111553

[112] Aranci K, Uzun M, Su S, Cesur S, Ulag S, Amin A, et al. 3D Propolis-sodium alginate scaffolds: influence on structural parameters, release mechanisms, cell cytotoxicity and antibacterial activity. Molecules. 2020. 10.3390/molecules25215082. 10.3390/molecules25215082 PMC7662765 33147742

[113] Batool S, Liaqat U, Hussain Z. Preparation and physicochemical characterization of whitlockite/PVA/Gelatin composite for bone tissue regeneration. Front Chem. 2024;12:1–10. 10.3389/fchem.2024.1355545. 10.3389/fchem.2024.1355545 PMC10900066 38420578

[114] Rasyida A, Halimah S, Wijayanti ID, Wicaksono ST, Nurdiansah H, Silaen YMT, et al. A composite of hydrogel alginate/PVA/r-GO for scaffold applications with enhanced degradation and biocompatibility properties. Polymers. 2023. 10.3390/polym15030534. 10.3390/polym15030534 PMC9921136 36771834

[115] Salati MA, Khazai J, Tahmuri AM, Samadi A, Taghizadeh A, Taghizadeh M, et al. Agarose-based biomaterials: opportunities and challenges in cartilage tissue engineering. Polymers. 2020;12:1–15. 10.3390/POLYM12051150. 10.3390/polym12051150 PMC7285176 32443422

[116] Wong RSH, Dodou K. Effect of drug loading method and drug physicochemical properties on the material and drug release properties of poly (ethylene oxide) hydrogels for transdermal delivery. Polymers. 2017. 10.3390/polym9070286. 10.3390/polym9070286 PMC6432290 30970963

[117] Aydin A, Ulag S, Sahin A, Aksu B, Gunduz O, Ustundag CB, et al. Biocompatible polyvinyl alcohol nanofibers loaded with amoxicillin and salicylic acid to prevent wound infections. Biomed Mater. 2023. 10.1088/1748-605x/acf25c. 10.1088/1748-605X/acf25c 37604153

[118] Khan FA, Narasimhan K, Swathi CSV, Mustak S, Mustafa G, Ahmad MZ, et al. 3D printing technology in customized drug delivery system: current state of the art, prospective and the challenges. Curr Pharm Des. 2019;24:5049–61. 10.2174/1381612825666190110153742. 10.2174/1381612825666190110153742 30636582

[119] Shi T, Liu Y, Wang D, Xia D, Li B, Xu R, et al. Spatially engineering tri-layer nanofiber dressings featuring asymmetric wettability for wound healing. Nano Mater Sci. 2024. 10.1016/j.nanoms.2024.01.008.

[120] Crabtree JR, Mulenga CM, Tran K, Feinberg K, Santerre JP, Borschel GH. Biohacking nerve repair: novel biomaterials, local drug delivery, electrical stimulation, and allografts to aid surgical repair. Bioengineering. 2024;11:776. 10.3390/bioengineering11080776 PMC11352148 39199733

[121] Shan Y, Xu L, Cui X, Wang E, Jiang F, Li J, et al. A responsive cascade drug delivery scaffold for peripheral nerve injury repair. Marer Horizons. 2024. 10.1039/d3mh01511d. 10.1039/d3mh01511d 38073476

[122] Moon YE, Jeong J-O, Choi H. Inflammation-responsive hydrogels in perioperative pain and wound management: design strategies and emerging potential. Gels. 2025;11:691. 10.3390/gels11090691 PMC12469436 41002466

[123] Karimzadeh Z, Namazi H. Nontoxic double-network polymeric hybrid aerogel functionalized with reduced graphene oxide: preparation, characterization, and evaluation as drug delivery agent. J Polym Res. 2022. 10.1007/s10965-022-02902-0.

[124] Trusek A, Kijak E. Drug carriers based on graphene oxide and hydrogel: Opportunities and challenges in infection control tested by amoxicillin release. Materials. 2021. 10.3390/ma14123182. 10.3390/ma14123182 PMC8228297 34207735

[125] Pooresmaeil M, Javanbakht S, Behzadi Nia S, Namazi H. Carboxymethyl cellulose/mesoporous magnetic graphene oxide as a safe and sustained ibuprofen delivery bio-system: synthesis, characterization, and study of drug release kinetic. Colloids Surfaces A Physicochem Eng Asp. 2020. 10.1016/j.colsurfa.2020.124662.

[126] Maşlakcı NN. Development and characterization of drug-loaded PVP/PAN/Gr electrospun fibers for drug delivery systems. ChemistrySelect. 2021;6:2548–60. 10.1002/slct.202004176.

[127] Duygulu NE, Altinbay A, Ciftci F. Antibacterial, mechanical, and thermal properties of Ag, ZnO, TiO_2_ reinforced PVA nanocomposite fibers. ChemistrySelect. 2024;9:e202402311. 10.1002/slct.202402311.

[128] Wibawa PJ, Nur M, Asy'ari M, Wijanarka W, Susanto H, Sutanto H, et al. Green synthesized silver nanoparticles immobilized on activated carbon nanoparticles: antibacterial activity enhancement study and its application on textiles fabrics. Molecules. 2021;26:1–14. 10.3390/molecules26133790. 10.3390/molecules26133790 PMC8270246 34206375

[129] Jokinen J, Dadu E, Nykvist P, Käpylä J, White DJ, Ivaska J, et al. Integrin-mediated cell adhesion to type I collagen fibrils. J Biol Chem. 2004;279:31956–63. 10.1074/jbc.M401409200. 15145957 10.1074/jbc.M401409200

[130] Zhang Q, Du Q, Zhao Y, Chen F, Wang Z, Zhang Y, et al. Graphene oxide-modified electrospun polyvinyl alcohol nanofibrous scaffolds with potential as skin wound dressings. RSC Adv. 2017;7:28826–36. 10.1039/c7ra03997b.

[131] Senthil R, Berly R, Ram TB, Gobi N. Electrospun poly(Vinyl) alcohol/collagen nanofibrous scaffold hybridized by graphene oxide for accelerated wound healing. Int J Artif Organs. 2018;41:467–73. 10.1177/0391398818775949. 29843552 10.1177/0391398818775949

